# Highly Charged Porphyrin Molecular Wires

**DOI:** 10.1021/jacs.6c09441

**Published:** 2026-08-31

**Authors:** Janko Hergenhahn, Sebastian M. Kopp, Edward R. Champness, Damyan Frantzov, Kavita Rani, Arzhang Ardavan, Harry L. Anderson, Christiane R. Timmel

**Affiliations:** † Centre for Advanced Electron Spin Resonance, Department of Chemistry, 6396 University of Oxford, Oxford OX1 3QR, U.K.; ‡ Chemistry Research Laboratory, Department of Chemistry, University of Oxford, Oxford OX1 3TA, U.K.; § The Clarendon Laboratory, Department of Physics, University of Oxford, Parks Road, Oxford OX1 3PU, U.K.

## Abstract

Successive oxidations, or reductions, of an extended molecular π-system can give rise to significant changes in the electronic structure compared to the neutral or singly charged counterparts. In this work, we investigated highly reduced states of butadiyne-linked porphyrin oligomers with up to nine porphyrin units and one negative charge per porphyrin. CW-EPR and ^1^H Mims ENDOR spectroscopy were used to probe the proton hyperfine interactions in these polyanions. Both techniques show a continuous narrowing of the spectra with simultaneously increasing charge and oligomer length corresponding to a decrease in the magnitudes of the largest hyperfine interactions. This is consistent with density functional theory calculations that support delocalization of spin density over the entire lengths of the molecules with high degrees of spin polarization and nonuniform spin density distributions. These spin distributions can also be described by a Hubbard model with antiferromagnetic spin coupling between electrons on adjacent sites. Finding systems with coherent wave functions that span the entire length of π-conjugated molecules, up to a length of more than 10 nm, will help identifying suitable candidates capable of coherent electron tunneling through molecular junctions.

## Graphical abstract

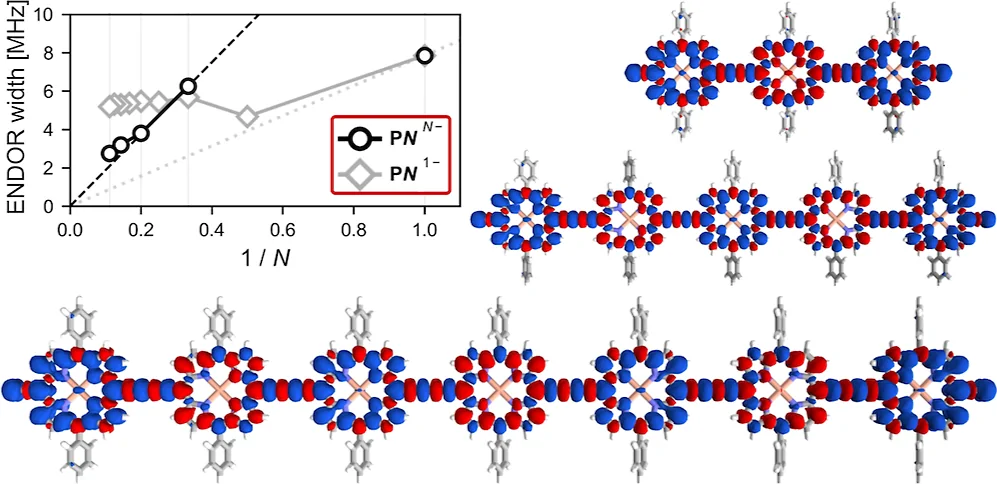

## Introduction

Conjugated porphyrin oligomers are extensively studied for their electronic properties and are promising candidates for nanoelectronic applications such as field-effect transistors and molecular wires.^1–5^ The spatial extent of the charge carriers influences the charge transport mechanism in molecular junctions, and long delocalization lengths are associated with high charge carrier mobilities and coherent charge transport.^6–8^ Introducing multiple charges into organic π-systems can give rise to significant changes in their electronic structure compared to the neutral or singly charged counterparts, and the charge states of porphyrin-based junctions have been shown to strongly affect their conductance.^9,10^ Furthermore, conjugated porphyrin nanorings have been shown to exhibit aromatic and antiaromatic ring currents in some oxidation states.^11,12^ The switch from local aromaticity (within porphyrin units) to global aromaticity (around the entire perimeter of the ring) in more highly charged states points toward a stronger delocalization of charge carriers and is a further motivation to investigate these states in detail.

While polycationic species have been investigated in various oligomeric systems,^13–15^ polyanionic π-systems have been less extensively studied. Most reported studies of polyanions involved relatively small systems such as isopyrenes,^16^ corannulenes,^17^ annulenes,^18,19^ and tetracyano-xylylene derivatives^20^ and due to the limited size of these molecules, the spin densities are unsurprisingly spread over the entire molecules.^21^ One study has investigated the polyanionic species in oligomers of increasing length in a family of oligo­[*N*]­cruciforms.^22^ Both the monoanions and the polyanions (i.e., one charge per repeat unit) had spin densities that were delocalized over the entire chain, although the polyanions had a higher nonuniformity, indicative of stronger electronic coupling between the subunits.^23^

Previously, the radical monoanions of a class of butadiyne-linked porphyrin oligomers **P** ***N*** (see Figure 1) have been studied in which the delocalization of spin density is limited to about four porphyrin units, with most of the spin density on just two units.^24^ Here, we show that further reduction of these oligomers results in more delocalized spin density distributions. CW-EPR and pulsed hyperfine spectroscopy are established tools to investigate the extent of delocalization in open-shell oligomeric systems^23,25–27^ and were used to probe the proton hyperfine interactions. Density functional theory (DFT) calculations provide detailed insights into the experimental trends and show that the highest reduced polyanion states (i.e., one charge per porphyrin) result in spin densities that are spread over the entire molecules.

**Figure 1:**
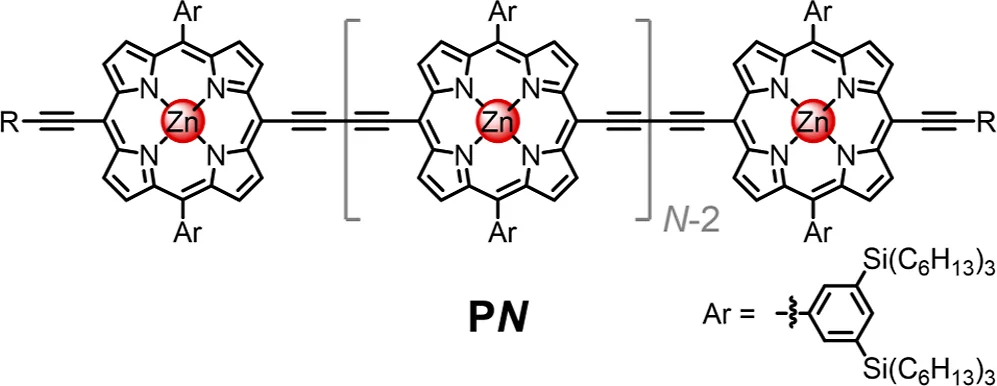
Structure of linear porphyrin oligomers **P** ***N*** (R = Si­(C_6_H_13_)_3_, *N* = 1, 3, 5, 7, or 9).

## Results and Discussion

### Room-Temperature CW-EPR

To investigate the EPR-spectra of different charge states, samples of various butadiyne-linked porphyrin oligomers **P** ***N*** (*N* = 3, 5, 7) were incrementally reduced by addition of the reducing agent decamethyl cobaltocene CoCp_2_^*^. The titrations started from the neutral oligomers and were continued until enough reducing agent was added to add one electron per porphyrin to obtain the highly reduced oxidation states **P** ***N*** ^***N–***^. Square wave voltammetry on **P** ***N*** and CoCp_2_^*^ were performed to confirm that CoCp_2_^*^ reduces the porphyrin oligomers up to once per porphyrin unit (see Supporting Information (SI), Figure S1). To avoid aggregation, porphyrin oligomers with trihexylsilyl (THS) side chains were used and all EPR measurements were done in THF with 10 mM tetrabutylammonium hexafluorophosphate (TBAP) electrolyte. This electrolyte was added to screen ion pairing effects.^24^ The reducing agent was added to the porphyrin solution under an inert atmosphere inside a glovebox, and samples were kept in airtight J Young EPR tubes. The samples were removed from the glovebox for EPR measurements between additions of reducing agent, and the anions produced this way gave rise to stable EPR signals over the time scale of the titration experiment (see SI, Figure S2). All steps were done at room temperature (298 K).

Addition of a reducing agent to **P3** leads to the formation of the known **P3** ^**1–**^ anion.^24^ The signal intensity increases until one equivalent of CoCp_2_^*^ is added, after which the signal intensity drops again with a minimum at two equivalents of reducing agent. Further additions result in a maximum of the signal intensity once three equivalents are added (see Figure 2a,b). No change in the signal shape was observed upon further addition of CoCp_2_^*^. These observations are consistent with a closed-shell dianion state and open-shell doublet monoanion **P3** ^**1–**^ and trianion **P3** ^**3–**^ anion states. While only the monoanion **P3** ^**1–**^ has been studied by EPR previously, all three reduction states have been observed in spectro-electrochemistry experiments,^24^ in which near-infrared (NIR) spectra were recorded while the potential was lowered until the porphyrin oligomer was reduced to a state corresponding to one charge per porphyrin. The resulting 2D-spectra were resolved into the individual spectra of **P3** ^***M–***^ (*M* = 1, 2, 3) by using multivariate curve resolution (MCR). Similarly, here we have used MCR to extract the CW-EPR signals of **P3** ^**1–**^ and **P3** ^**3–**^ from the reduction titration series as shown in Figure 2e (see Supporting Information for details). The CW-EPR signal of **P3** ^**3–**^ has no resolved hyperfine pattern and a smaller *g*_iso_-value compared to **P3** ^**1–**^ (2.0011 compared to 2.0017; simulations of the spectra are shown in the SI, Figure S4).

**Figure 2:**
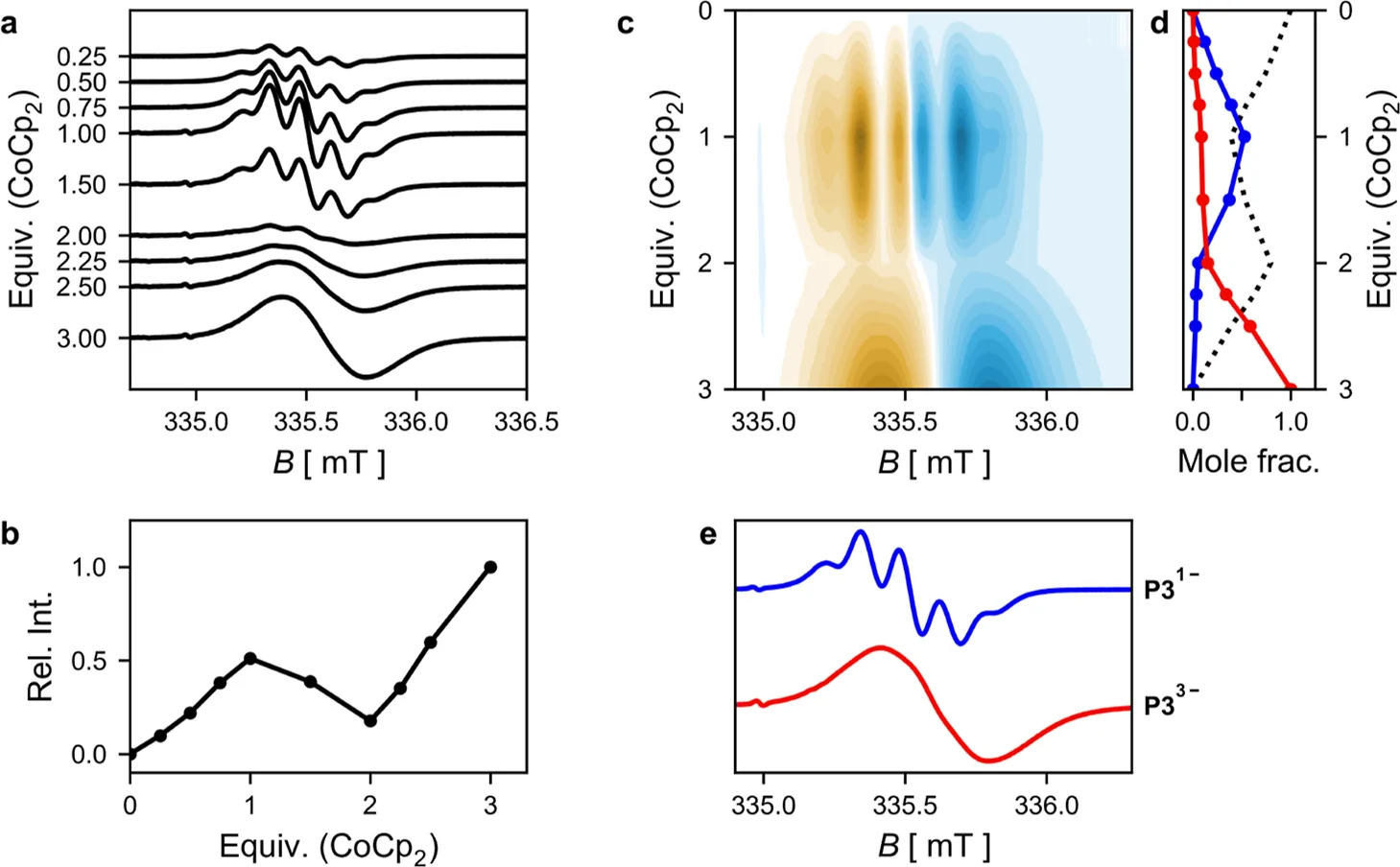
(a) CW-EPR spectra of **P3** recorded in THF with 10 mM TBAP at 298 K and X-band frequency (9.40 GHz) with increasing amounts of reducing agent CoCp_2_^*^. (b) Signal intensity during the titration determined from the double integral (after background correction). (c) 2D-map of the reduction titration. (d) Relative concentrations and (e) normalized, deconvoluted CW-EPR spectra of the species present in the reduction titration. The colors and labels in plot (e) correspond to the speciation curves in plot (d), and the dotted line in plot (d) shows an estimate of the concentration of EPR-silent species.

Deconvolution of the EPR spectra by MCR provides relative concentrations of the EPR-active species **P3** ^**1–**^ and **P3** ^**3–**^ (Figure 2d). Electrochemistry measurements show that CoCp_2_^*^ can achieve full conversion of **P3** to the trianion (see SI, Figure S1), and therefore the relative concentration of **P3** ^**3–**^ can be assumed to be 1.0 at the final point of the reduction titration. In contrast, the maximum relative concentration of the monoanion is only about half that of the trianion because of overlapping adjacent reduction potentials (see Figure 2d), which mean that **P3** ^**1–**^ cannot be produced in isolation from its adjacent (EPR-silent) oxidation states. The MCR analysis does not rely on the presence of any EPR-silent species, and their individual relative concentrations can consequently not be extracted. However, the total relative concentration of EPR-silent species can still be estimated from the MCR results by subtracting the total relative concentration of EPR-active species from an overall relative concentration of unity (black dotted line in Figure 2d). The resulting concentration profile is consistent with the presence of diamagnetic neutral **P3** and **P3** ^**2–**^.

The titration of **P5** results in three maxima and two minima in the EPR signal intensity (see Figure 3a) consistent with open-shell doublet states for **P5** ^**1–**^, **P5** ^**3–**^, and **P5** ^**5–**^ and closed-shell singlet states for **P5** ^**2–**^ and **P5** ^**4–**^. The minima still have considerable signal intensity, which is probably due to the increasingly closely spaced reduction potentials in the longer oligomers (see electrochemistry measurements in SI, Figure S1). The multiply charged species of **P5** have been observed previously by NIR spectro-electrochemistry.^24^ The EPR signals of the open-shell species were obtained from MCR analysis of reduction titration (see Figure 3b). Singular value decomposition was attempted to estimate the number of components in the titration to test whether, for example, **P5** ^**4–**^ should be considered to be open shell and included in the simulations, but due to the similarity of the signals, no clear-cutoff could be determined. Therefore, only the three components, which can be clearly seen from the three maxima in signal intensity, were used for the analysis. While this introduces some amount of uncertainty over the spectral shape of **P5** ^**3–**^, the spectrum of **P5** ^**5–**^ is obtained with high confidence as the end-point of the reduction titration given the full conversion to the **P** ***N*** ^***N–***^ polyanion state. There are no resolved hyperfine patterns in the three deconvoluted spectra but there is a continuous decrease in *g*_iso_-value between the three signals (2.0017, 2.0013, and 2.0009 for **P5** ^**1–**^, **P5** ^**3–**^, and **P5** ^**5–**^, respectively).

**Figure 3:**
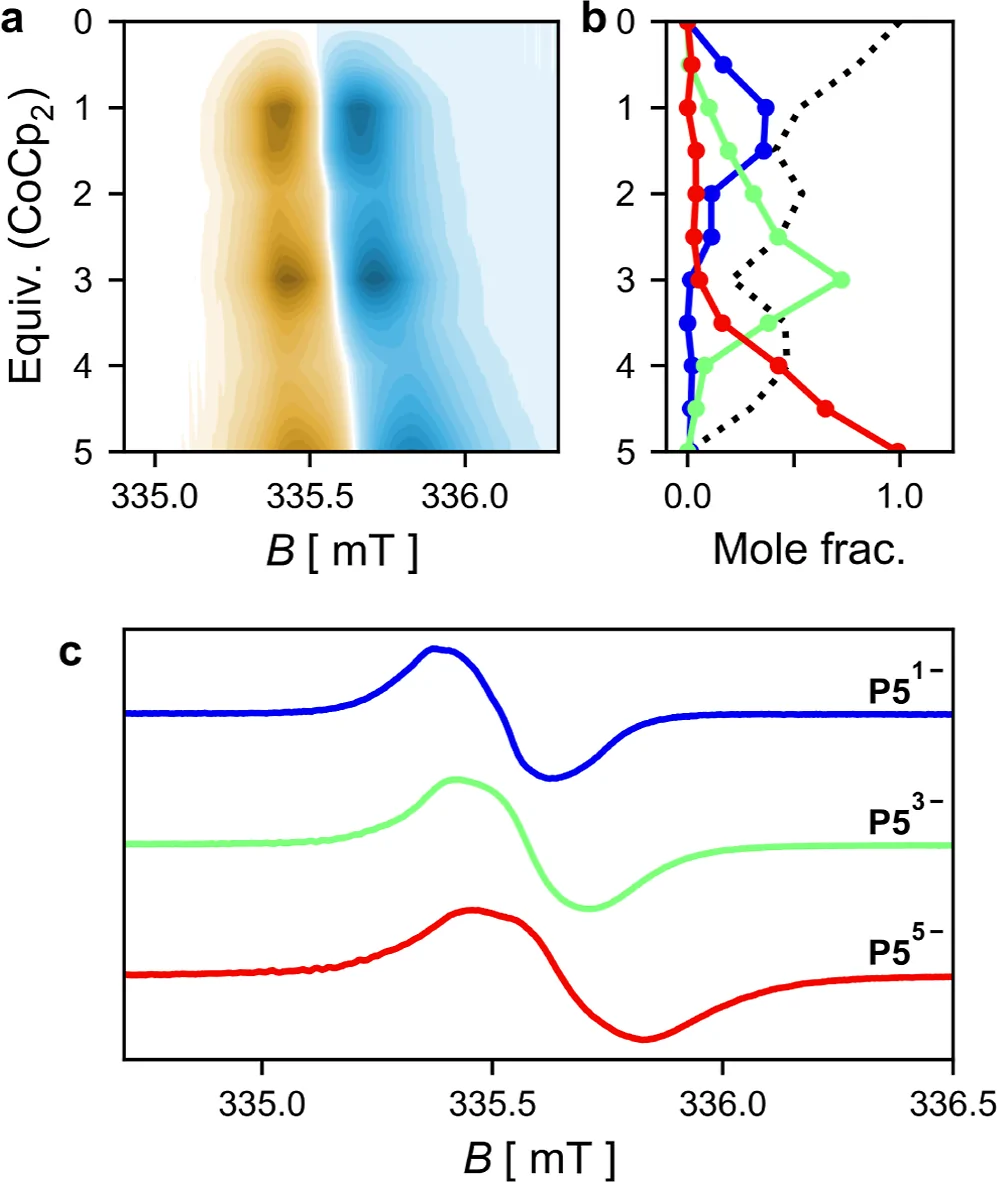
(a) 2D-map of CW-EPR spectra from the reduction titration of **P5** recorded in THF with 10 mM TBAP at 298 K and X-band frequency (9.40 GHz) with increasing amounts of reducing agent CoCp_2_^*^. (b) Relative concentrations and (c) normalized, deconvoluted CW-EPR spectra of the species present in the reduction titration. The colors and labels in plot (c) correspond to the speciation curves in plot (b), and the dotted line in plot (b) shows an estimate of the concentration of EPR-silent species.

Based on the trends seen in **P3** and **P5**, the titration of **P7** was expected to show four distinct maxima. However, the signal intensity during the titration has only three maxima with a strong peak at about two equivalents (see Figure 4a). A slight excess (8 equiv) was added to reach the final point of the titration, which is likely due to the highest reduction potential of **P7** being similar to that of the reducing agent CoCp_2_^*^. Deconvolution of the reduction titration data was attempted for different numbers of components (see SI, Figure S3), and the best results were obtained when using five components as shown in Figure 4c. While the deconvolution introduces some uncertainty about the EPR spectral shapes of the intermediate charge states, the EPR spectrum of the heptaanion **P7** ^**7–**^ can be obtained with good confidence by the addition of a slight excess of reducing agent. The concentration profiles resulting from the MCR analysis indicate that the dianion **P7** ^**2–**^ has an open-shell character, while the tetraanion **P7** ^**4–**^ and hexaanion **P7** ^**6–**^ remain closed shell. The spectrum assigned to **P7** ^**2–**^ is wider than that of the monoanion **P7** ^**1–**^, possibly due to the additional intramolecular dipolar interaction between electrons. As in the other systems, there is a continuous shift in the *g*_iso_-value for increasingly reduced oligomer **P7** (2.0017, 2.0015, 2.0011, 2.0007 for 1-, 3-, 5-, 7-, respectively). Due to a lack of features, simulation of the spectra did not provide further insights into other interactions (see Supporting Information, Figure S4).

**Figure 4:**
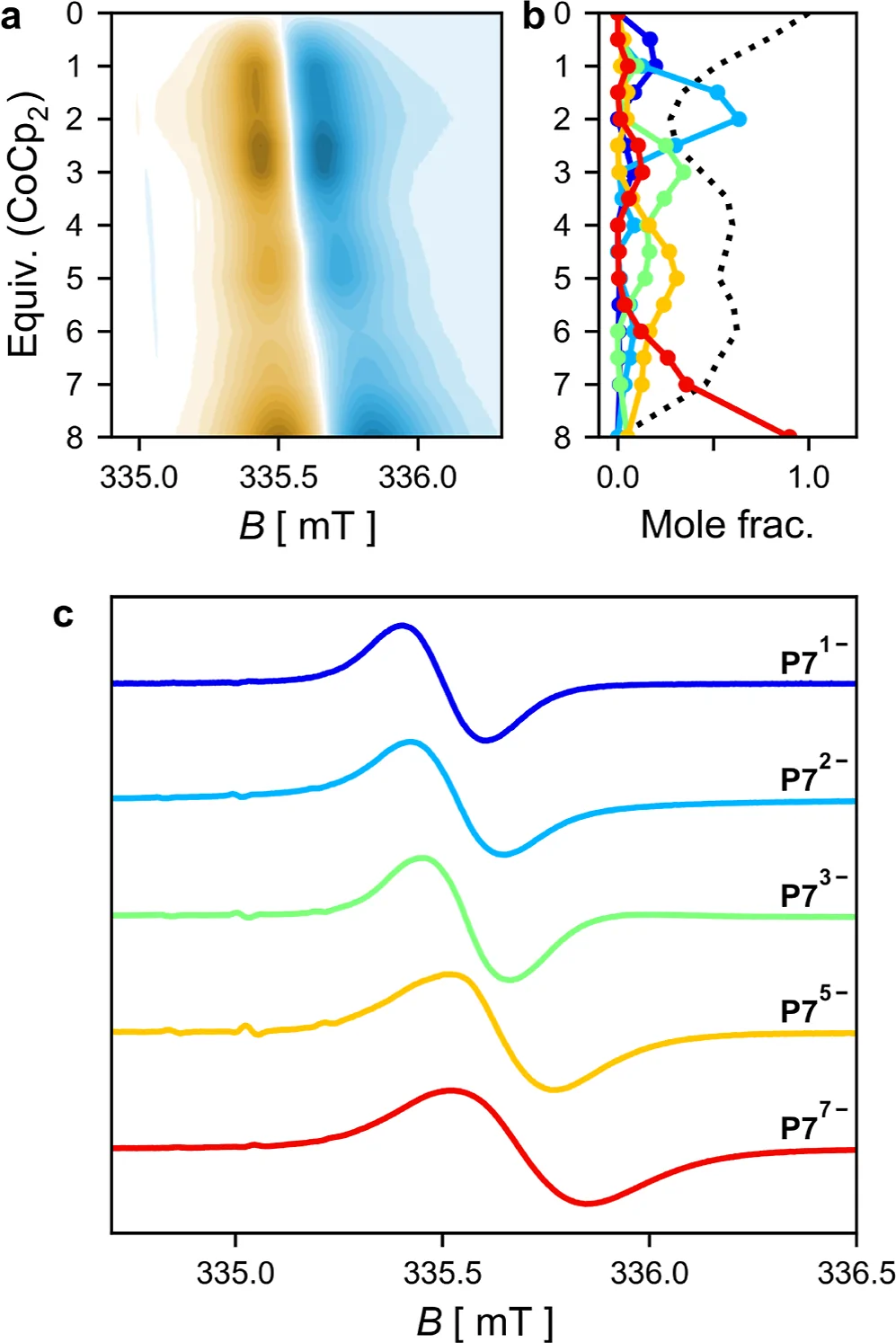
(a) 2D-map of CW-EPR spectra from the reduction titration of **P7** recorded in THF with 10 mM TBAP at 298 K and X-band frequency (9.40 GHz), with increasing amounts of reducing agent CoCp_2_^*^. (b) Relative concentrations and (c) normalized, deconvoluted CW-EPR spectra of the species present in the reduction titration. The colors and labels in plot (c) correspond to the speciation curves in plot (b), and the dotted line in plot (b) shows an estimate of the concentration of EPR-silent species.

Due to the closely spaced reduction potentials, it is not possible to produce arbitrary oxidation states *M* of the oligomers in pure form (**P** ***N*** ^***M–***^) except when *M* = 0 or *M* = *N*. Therefore, the analysis of the spin density delocalization will focus on the highest reduced state (*M* = *N*) of the odd-numbered oxidation states **P** ***N*** ^***N–***^ (*N* = 1, 3, 5, 7), which can be obtained cleanly by adding *N* equivalents (or a slight excess) of reducing agent as shown above. This restricts the analysis to the odd-numbered oligomers as the even numbered systems **P** ***N*** ^***N–***^ (*N* = 2, 4, 6) would be closed-shell species.

The CW-EPR spectra of **P** ***N*** ^***N–***^ (*N* = 3, 5, 7) have an approximately Gaussian derivative line shape due to the large number of unresolved hyperfine coupling interactions. The delocalization in such systems has been interpreted in terms of the Norris equation in the past,^25,28^ which states that for complete and uniform delocalization of spin density, the widths of the spectral envelopes of the CW-EPR spectra vary linearly with $\frac{1}{\sqrt{N}}$ :

1 $$\sigma_{N}=\frac{\sigma_{N=1}}{\sqrt{N}}$$

where *N* is the number of sites in an oligomer and σ_*N*=1_ is the width of the monomer. Apart from complete and uniform delocalization, this relationship requires the absence of any additional line-broadening effects.^26^ In order to account for other broadening contributions, the room-temperature CW-EPR spectra were fitted using Voigtian line shape profiles including Gaussian and Lorentzian contributions. The Gaussian and Lorentzian components σ and γ of the line shapes of **P** ***N*** ^***N–***^ (*N* = 3, 5, 7) obtained from this fitting procedure are shown in Figure 5b,c, respectively, along with the line shapes of their respective monoanions.

**Figure 5:**
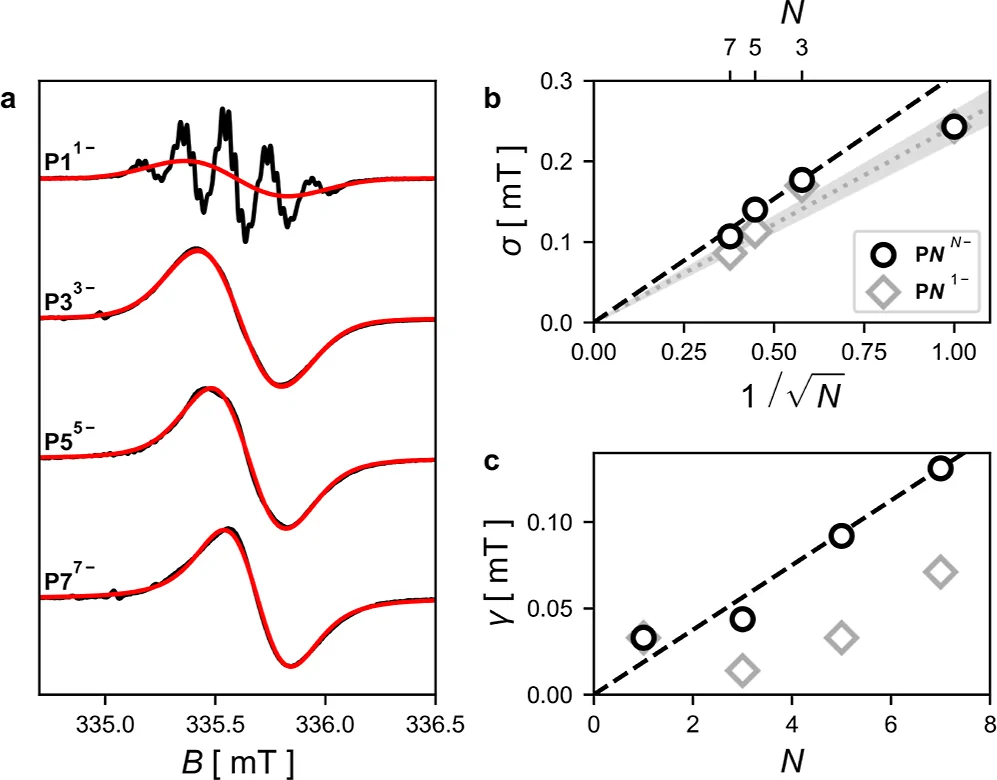
(a) CW-EPR spectra of **P** ***N*** ^***N–***^ (*N* = 1, 3, 5, and 7) recorded at 298 K in THF with 10 mM TBAP at X-band frequencies (black) and fitted Voigitan derivative spectral envelopes (red). (b) Gaussian components of the fitted Voigtian profiles. (c) Lorentzian components of the fitted Voigtian profiles. Line shape parameters of the monoanionic systems **P** ***N*** ^***N–***^ are shown in gray for comparison. Note that **P1** ^**1–**^ belongs to both data sets.

While the Gaussian components σ of the CW-EPR spectra form a straight line for the polyanions **P** ***N*** ^***N–***^ for *N* = 3, 5, and 7, the monomer **P1** ^**1–**^ does not fit the same trend. Note that the Norris trend line is in principle completely defined by the monomer data point as it must also pass through the origin. This means that all longer oligomers have a larger Gaussian component than would be expected from the Norris equation when considering the spin density of **P1** ^**1–**^ as the repeat unit of the polyanionic systems. Therefore, this indicates either incomplete delocalization or nonuniformity of the spin density in these systems.^26^ The continuous decrease in the Gaussian line width suggests that there may be further delocalization in the longer oligomers and the origin of the deviations will be explored further by also considering frozen solution ^1^H ENDOR measurements and DFT calculations shown below. The Lorentzian components, on the other hand, show a linear increase with oligomer length. This could be a result of a slower rotational correlation time for longer oligomers, as well as their larger *g*-anisotropy (compared to the monoanions). The latter is reflected in the lower *g*_iso_ values of the polyanionic systems and confirmed by the pulsed EPR measurements done in frozen solution.

### Pulsed EPR Measurements

Low-temperature EPR measurements on polyanionic systems in frozen solution can provide additional insights into the extent of delocalization of the spin density in the absence of dynamic effects. Again, the focus was on the highest oxidation states of the odd-numbered linear oligomers **P** ***N*** ^***N–***^ (*N* = 1, 3, 5, 7, 9), which were obtained by adding an excess (1.5x *N* equivalents) of the reducing agent CoCp_2_^*^. These polyanions have similar EPR-spectra to the previously studied monoanions,^24^ with no resolved hyperfine pattern and an axial *g*-tensor (see SI, Figure S5). The *g*-anisotropies in **P** ***N*** ^***N–***^ are larger than in **P** ***N*** ^***N–***^ and, in particular, their *g*_∥_ component extends to larger field values (lower *g*-values). Phase-inverted echo-amplitude detected nutation (PEANUT) experiments,^29^ which have previously been used to elucidate the spin state in porphyrin anion systems,^30^ confirm that the polyanions **P** ***N*** ^***N–***^ exist in a doublet state (see SI, Figure S6).

In order to get more detailed insights into the hyperfine coupling interactions in **P** ***N*** ^***N–***^, ^1^H ENDOR spectra were recorded at 80 K for *N* = 3, 5, 7, and 9 as shown in Figure 6a along with the respective spectra of the monoanions (note that for monomer **P1,** the monoanion and polyanion are the same species). While the trianion of the trimer **P3** ^**3–**^ has a very similar spectral shape and width compared to the monoanion **P3** ^**1–**^, the ^1^H ENDOR spectra of the polyanionic **P** ***N*** ^***N–***^ (*N* = 5,7,9) are noticeably narrower than for their monoanionic counterparts, which means they have a smaller dominating hyperfine coupling.

**Figure 6:**
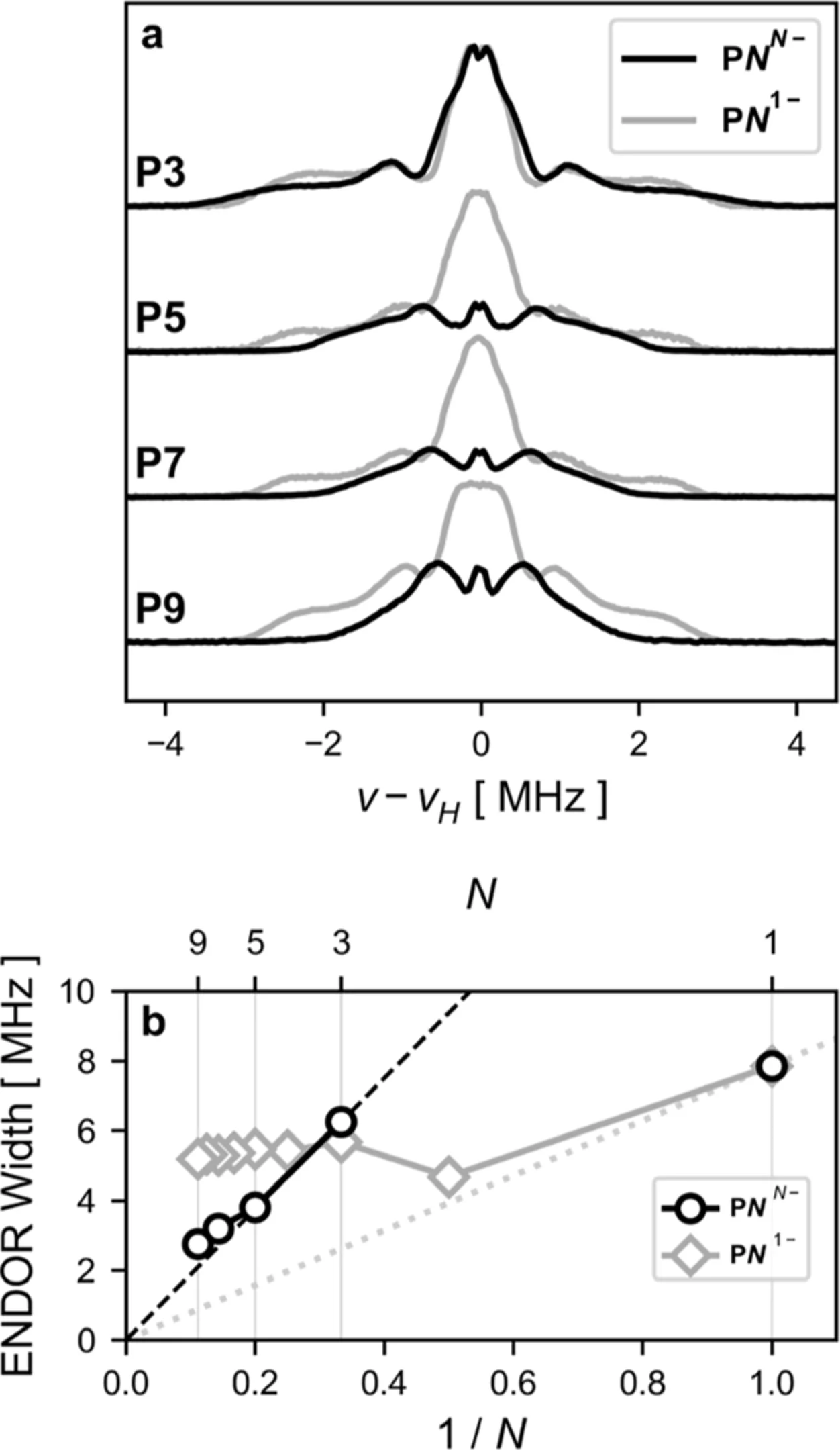
(a) ^1^H ENDOR spectra of **P** ***N*** ^***N–***^ (*N* = 3, 5, 7) recorded at field positions corresponding to *g*_⊥_ at 80 K at Q-band frequencies averaged over four τ values (τ = 150, 210, 270, and 330 ns). (b) Trend in ENDOR spectral widths. The dotted line shows the trend for full and uniform delocalization, and the shaded area indicates a 95% confidence interval. Data for the monoanions are shown for comparison.

Due to the shape of these spectra, their widths cannot be simply extracted from their full width at half-height and were instead determined by finding the first and last inflection points (see Supporting Information, Figure S8). The ^1^H ENDOR widths obtained this way are shown in Figure 6b and show a linear trend for **P** ***N*** ^***N–***^ (*N* = 3, 5, 7, 9) that extrapolates to the origin. The trend of the ^1^H ENDOR spectral widths of the polyanions **P** *N* ^***N–***^ is therefore consistent with the trend of the Gaussian components obtained from the Voigtian profile fitting of the room-temperature CW-EPR spectra, including the fact that the monomer **P1** ^**1–**^ does not fall on the same trend line as **P** ***N*** ^***N–***^ (*N* = 3,5,7). This is in stark contrast to the previously studied monoanions **P** ***N*** ^***N–***^, in which the ^1^H ENDOR spectra were nearly identical for *N* ≥ 4, which indicated the limit of the delocalization.^24^ A further narrowing of the spectral envelopes in the room-temperature CW-EPR spectra showed that in those systems dynamic effects lead to a further (noncoherent) spread of spin density on the EPR-time scale. In contrast, the matching trends in the polyanion systems **P** ***N*** ^***N–***^ and the further narrowing of the ^1^H ENDOR spectra even up to *N* = 9 therefore suggests that the deviation from an ideal Norris trend is likely to result from the nonuniformity of the spin density rather than from incomplete delocalization. This is further supported by the DFT calculations shown below.

### Spin Density Modeling and Simulation

DFT calculations were used to model the spin densities of the polyanions **P** ***N*** ^***N–***^ (*N* = 3, 5, 7). Structures of the porphyrin oligomers were optimized at the PBE0+D3BJ level of theory with def2-SVP­(H, C, N)+def2-TZVP­(Zn) basis sets using Gaussian 16 with a tight convergence threshold.^31–33^ All calculations were done with phenyl side chains instead of 3,5-bis­(THS)­phenyl to improve computational speed. Calculations of EPR parameters on the optimized structures were subsequently carried out in ORCA 5.0.^34^ Initially, calculations on **P** ***N*** ^***N–***^ were done with the range-separated functional lc-ωPBE (ω = 0.175)^35^ that had previously been found to give the best description of the spin densities in the monoanionic systems^24^ but resulted in much too large hyperfine coupling interactions compared with experiment (see SI, Figure S10). This is probably due to an incorrect characterization of spin polarization, which is necessary to correctly describe hyperfine coupling interactions, particularly in systems in which the unpaired electron is in a π-orbital,^36,37^ and strongly depends on the choice of exchange–correlation functional.^38^ The DFT functional was systematically altered to obtain a series of different spin density distributions for the polyanions **P** ***N*** ^***N–***^ (*N* = 3, 5, 7). All calculations were based on the PBE exchange and correlation functionals^39^ but with different contributions of exact exchange. This was controlled with the range-separation parameter ω and the parameter α determining the constant amount of exact exchange that is added to the functional. The best fit to experimental spectra was obtained for α = 0.1 and a small ω of about 0.01–0.05 (see Supporting Information, Figures S9 and S10). The combination of a small, constant exact-exchange term with a small range-separation constant has also recently been found to give a good description of other porphyrin systems.^40^

All DFT calculations on the polyanions **P** ***N*** ^***N–***^ (*N* = 3, 5, 7) resulted in spin densities that were spread over the entire length of the oligomers. The spin densities resulting in hyperfine coupling constants that gave the best agreement with the experimental spectra are shown in Figure 7 along with the simulated ^1^H ENDOR spectra. The spectra were calculated using *EasySpin* ^41^ and were obtained by adding together simulations of systems with one electron and one nucleus for each hyperfine coupling considered. Only contributions from β pyrrole hydrogen atoms of the porphyrins were used as they give rise to the characteristic shoulders of the porphyrin anion ^1^H ENDOR spectra, while the central regions mainly arise from weak hyperfine couplings, e.g., to the hydrogen atoms of the solubilizing side chains.

**Figure 7:**
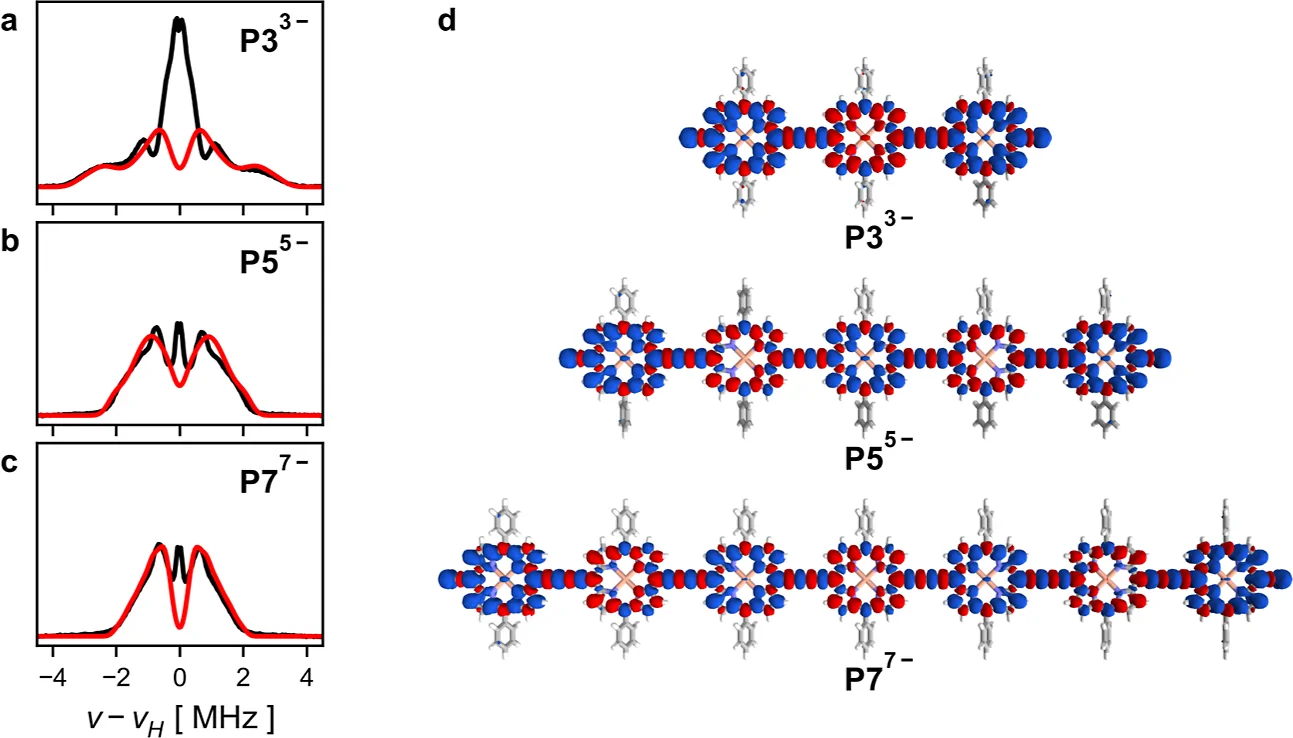
(a–c) Experimental ^1^H ENDOR spectra of **P** ***N*** ^***N–***^ (black) and simulation (red) based on DFT-calculated hyperfine coupling tensors. (d) Spin densities of **P** ***N*** ^***N–***^ that resulted in hyperfine coupling constants with best fit to experimental spectra; lc-ωPBE with (α = 0.1, ω = 0.05) for **P3** ^**3–**^ and (α = 0.1, ω = 0.01) for **P5** ^**5–**^ and **P7** ^**7–**^. Inset: third, fifth, and seventh lowest wave functions for a particle-in-a-box model.

The spin densities of **P** ***N*** ^***N–***^ have an interesting nodal pattern with an alternation between positive and negative phases (between α and β spin densities) from porphyrin to porphyrin. Considering the alternating phase pattern of the spin density, it is clear that the Norris equation cannot accurately describe the expected spectral width in the polyanion oligomers **P** ***N*** ^***N–***^ due to the high nonuniformity of the spin density. This nonuniformity explains the deviation that was seen in the spectral widths of the ^1^H ENDOR spectra: the alternating phase pattern resulting from regions with large spin polarization leads to a slower decrease in the maximum spin density at a given porphyrin unit and therefore a slower decrease in the maximum hyperfine coupling constant than predicted by the Norris equation. Figure 8 shows that the maximum spin densities on a porphyrin unit form a linear trend for **P** ***N*** ^***N–***^ (*N* = 3, 5, 7) that is different from the trend expected for complete and uniform delocalization. This trend closely resembles the one observed for the widths of the ^1^H ENDOR spectra (see Figure 6) and explains why the monomer **P1** ^**1–**^ does not fit the same trend as the longer systems.

**Figure 8:**
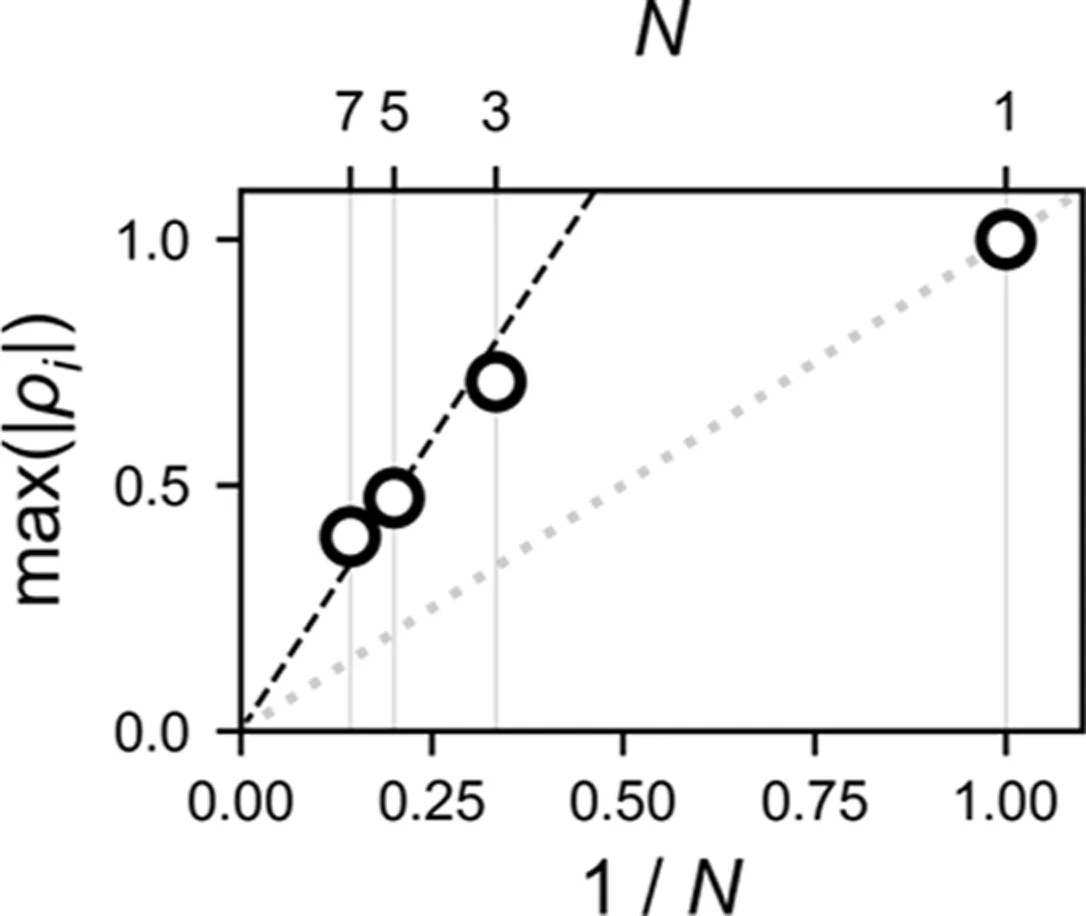
Largest spin density population on a single porphyrin for different polyanion oligomers **P** ***N*** ^***N–***^ in the spin density that gave the best fit to experimental ^1^H ENDOR spectra. The gray dotted line indicates the trend expected for complete and uniform delocalization.

It was noted that even for the spin density distributions that resulted in good agreement with the experimental spectra, there were still large amounts of spin contamination present in the wave functions. Some extent of “spin-contamination” is required in any unrestricted Kohn–Sham wave function in order to describe spin polarization, as deviations away from a restricted open-shell character wave function mean it is no longer an eigenfunction of the total spin operator ${Ŝ}^{2}$ .^42^ However, large values of spin-contamination and spatial separation of alpha and beta Kohn–Sham spin orbitals (as found in the wave functions for **P** ***N*** ^***N–***^, see SI Figure S13) probably indicate an admixture of higher multiplicity wave functions and therefore a multireference character of the Kohn–Sham determinant, which cannot be fully captured by single-determinant DFT calculations.^43^ In order to capture the full physics of these systems, multideterminant calculations such as CASSCF would be necessary,^43,44^ but the size of the these systems unfortunately makes these calculations not feasible. However, EPR experiments showed that the **P** ***N*** ^***N–***^ polyanions indeed behave as doublets, and therefore the spin contamination and spatial separation of alpha and beta spin are probably a sign of a system with strong antiferromagnetic coupling between unpaired electron spins.

Integration of the spin density along the long axis of the porphyrin chain results in a phase pattern reminiscent of the wave functions for the particle-in-a-box model as shown in Figure 9a,b. However, particle-in-a-box is a noninteracting model, which cannot give the full picture in a many-electron situation. Electron repulsions are likely to play a large role in highly charged systems like the **P** ***N*** ^***N–***^ polyanions. Hence, we tried to rationalize our DFT results within the framework of the Hubbard model—one of the cornerstone models in condensed matter physics dealing with interacting electrons.^45–47^ The model consists of *M* electrons hopping on *N* lattice sites. In our case, the porphyrin oligomer is modeled as a 1D linear chain, with each site representing a porphyrin unit in the oligomer. The Hamiltonian governing this system is given below in the second quantization notation, i.e., written on the basis of possible electron configurations

$$Ĥ=U\sum_{i}c_{i,\alpha }^{†}c_{i,\alpha }c_{i,\beta }^{†}c_{i,\beta }-t\sum_{\left\langle ij\right\rangle ,\sigma }c_{i,\sigma }^{†}c_{j,\sigma }$$

where the *c*_*i*,σ_^†^ (*c*_*i*,σ_) are the Fermionic creation (destruction) operators for electrons on site *i* with spin σ. The first term describes the on-site repulsion when electrons occupy the same site, parametrized by the on-site repulsion term *U*. The second term describes the stabilization due to hopping of electrons to neighboring sites where *t* is a kinetic energy term and takes on a similar role to resonance in Hückel theory. In a 1D-system where *U* ≪ *t*, the Hubbard model predicts metallic conductivity. In contrast, a large on-site repulsion term (*U* ≫ *t*) leads to a system with one electron on each site and antiferromagnetic spin coupling to electrons on adjacent sites (Mott insulator). Such a system would result in a spin pattern similar to that observed in the **P** ***N*** ^***N–***^ anions but with a spin density of one per site. A Hubbard model in which *U*/*t* = 3.8 results in spin density distributions for the **P** ***N*** ^***N–***^ (*N* = 3,5,7) polyanions in good agreement with those obtained from DFT calculations (see Figure 9c). A similar magnitude of *U* and *t* indicates a state in which the electrons are delocalized but cannot move freely through the system and partially localize due to the repulsion from other electrons.^48^ While individual electrons are not coherently delocalized over the entire molecules, the extent of spin density over the entire molecule and its alternating α/β pattern obtained from both DFT and a simple Hubbard model are consistent with long-range spin coherence and strong antiferromagnetic correlations between electrons.

**Figure 9:**
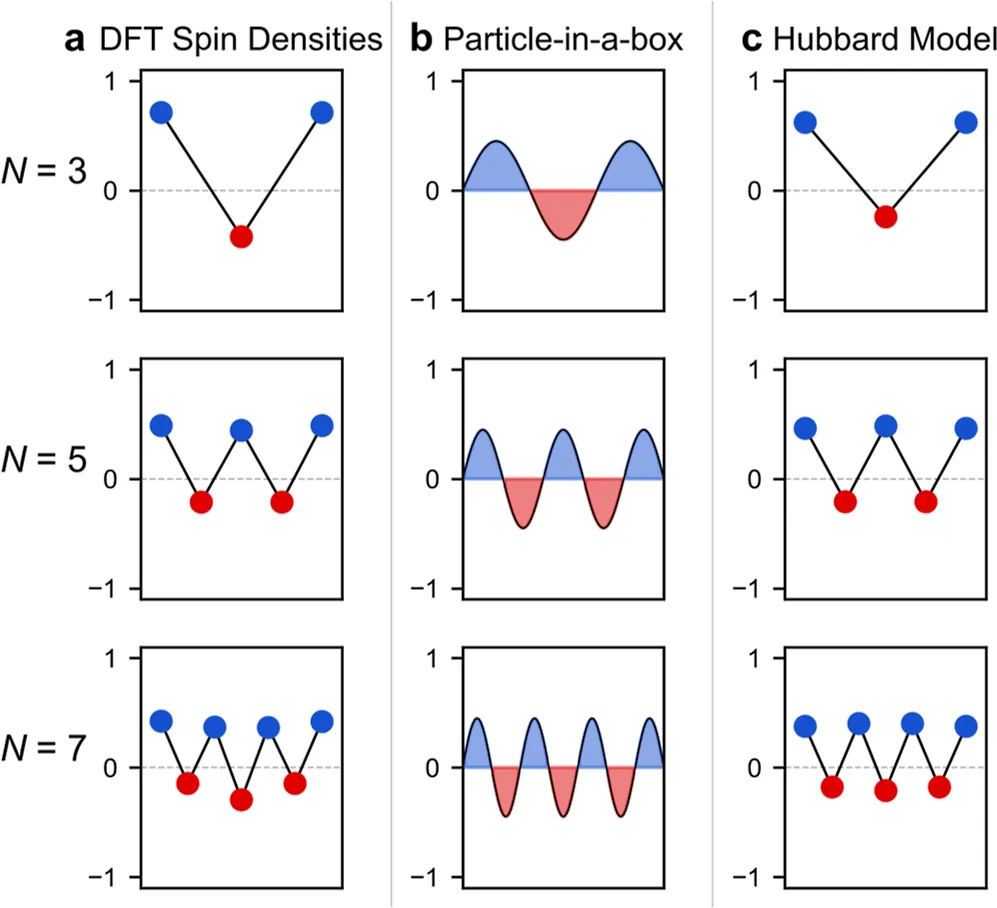
(a) Spin density distribution evaluated from DFT calculations integrated over each porphyrin site for **P** ***N*** ^***N–***^ (*N* = 3, 5, 7). (b) Particle-in-a-box wave functions for *N* = 3, 5, 7. (c) Hubbard model for *N* electrons distributed over *N* sites (*N* = 3, 5, 7) with *U*/*t* ≈ 3.8.

## Conclusion

In this work, multiply reduced butadiyne-linked porphyrin oligomers were investigated by CW-EPR, pulsed EPR and DFT calculations. In the shorter oligomers, even-numbered oxidation states were found to be EPR-silent while the odd-numbered oxidation states gave rise to doublet states. Both room-temperature CW-EPR and low-temperature frozen-solution ^1^H ENDOR spectroscopy of the polyanions **P** ***N*** ^***N–***^ show a continuous narrowing of the spectral widths, consistent with DFT calculations that show delocalization of the spin density over the entire length of the molecules.

The spin densities in **P** ***N*** ^***N–***^ have alternating phases for successive porphyrins, which can be reproduced with a simple Hubbard model. DFT calculations and the Hubbard model indicate that the additional electrons are partially localized to porphyrins along the chain but extend to neighboring porphyrins to some degree. This results in strong antiferromagnetic coupling between electrons of adjacent sites and in spin coherence over the entire length of the molecule.

For the nonamer nonaanion **P9** ^**9–**^, the delocalization over the whole length of the molecule represents a coherence distance of about 12.2 nm, which is longer than the longest reported intramolecular delocalization length of a single charge carrier of about 8.5 nm in the radical cations of edge-fused porphyrin nanoribbons.^23^ While there is spin coherence over the entire molecule like in those previously studied systems, it is uncertain if charge transport will be similarly efficient in **P** ***N*** ^***N–***^ as charges probably cannot move independently. Still, the extent of delocalization of the spin density is remarkable considering that it was limited to only about three to four porphyrins in the corresponding monoanionic systems.^24^ This highlights the effectiveness of simple redox chemistry in modifying the electronic structure of large conjugated systems. In future, it will be interesting to study cyclic versions of these odd-numbered polyanions, in which antiferromagnetic coupling between electrons on neighboring porphyrins would lead to spin frustration.

## Supplementary Material



## Notes

Details on spectroscopy, theoretical calculations, data analysis, and simulation methods (PDF)
