# Supplementary material for: Highly Charged Porphyrin Molecular Wires
Source: J Am Chem Soc. 2026 Aug 31;148(36):38820–8. doi: 10.1021/jacs.6c09441 (PMC13587665; doi:10.1021/jacs.6c09441)
Supplement: Supplementary file 1 [file ja6c09441_si_001.pdf]

Supporting Information

Highly Charged Porphyrin Molecular Wires

Janko Hergenhahn,[a,b] Sebastian M. Kopp,[a,b] Edward R. Champness,[b] Damyan Frantzov,[b] Kavita Rani,[b] Arzhang Ardavan,[c] Harry L. Anderson*,[b] and Christiane R. Timmel,*,[a,b]

[a] Centre for Advanced Electron Spin Resonance, Department of Chemistry, University of Oxford, Oxford OX1 3QR, UK
[b] Chemistry Research Laboratory, Department of Chemistry, University of Oxford, Oxford OX1 3TA, UK
[c] Department of Physics, University of Oxford, The Clarendon Laboratory, Parks Road, Oxford OX1 3PU, UK

Contents

1. Methods........ 2
   1.1 Experimental Methods 2
   1.2 EPR Measurements 2
   1.3 Deconvolution of Reduction Titrations 3
   1.4 Computational Methods 3
2. Electrochemistry 5
3. CW-EPR Signal Stability 5
4. Optimal number of components for **P7** reduction titration 6
5. CW-EPR spectral simulations 7
6. Additional Pulsed EPR Data........ 8
7. Additional Data for Density Functional Theory Calculations 12
8. References 15

Supplementary computational data for this article are available at Zenodo, https://doi.org/10.5281/zenodo.21792526, including calculated *xyz* coordinates, DFT hyperfine coupling tensors and DFT output files for the polyanions $\mathbf{P3}^{3-}$, $\mathbf{P5}^{7-}$ and $\mathbf{P7}^{7-}$.

1. Methods

1.1 Experimental Methods

The butadiyne-linked porphyrin oligomers were synthesized as reported previously.[1] All samples for EPR measurements were prepared in THF with 0.1 M tetrabutylammonium hexafluorophosphate (TBAP) electrolyte under an inert nitrogen atmosphere inside a glove box (MBraun LabStar Glovebox). The one-electron reducing agent $CoCp^*_2$ was sublimed on a Schlenk line prior to cycling it into the glove box where d_8-tetrahydrofuran (THF) was added to make an approximately 10 mM solution. The concentration of the solution was then validated on an aliquot of the solution by quantitative NMR with t-butyl benzene as a reference. Samples for room-temperature CW-EPR measurements were prepared in 3.8 mm X-band EPR tubes with a J.Young cap. Reduction titrations were carried out by incremental additions of between 0.25 and 0.5 equivalents of $CoCp^*_2$ to the porphyrin solution. Each addition of reducing agent was done under inert atmosphere inside a glove box. Samples for pulsed EPR measurements were prepared in 1.8 mm Q-band tubes which were sealed with vacuum grease and shock-frozen in liquid N_2 after cycling them out of the glove box. No further additions of reducing agent are possible to these samples once they thaw, as oxygen can diffuse into the sample. Therefore, all Q-band samples were made by addition of the required amount of reducing agent as one addition (0.5 equivalents for measurements of the monoanion and an excess of 1.5 × N for measurements of the polyanion).

1.2 EPR Measurements

CW spectra were recorded at X-band frequencies on a Bruker Biospin EMX spectrometer with a high sensitivity Bruker probe head at room temperature. Modulation amplitudes were adjusted for the first spectrum of a series until the smallest features could be resolved and no longer changed in shape and ranged between 0.005 mT and 0.02 mT. Similarly, powers were adjusted until no broadening effects were observed. The parameters were then kept constant for the remaining spectra of a titration series.

Spin-spin relaxation times (T_m) were measured using 2-pulse echo decay experiments with the pulse sequence $\pi/2 - \tau - \pi/2 - \tau - echo$ with pulse lengths of $t_{\pi/2}$ = 16 ns and t_π = 32 ns. The delay time τ was varied between 180 ns and about 16000 ns in steps of 16 ns.

Pulsed EPR measurements were carried out at 80 K at Q-band frequencies using a Bruker E580 spectrometer with a Bruker EN 5107D2 resonator. Echo-detected field-sweeps were recorded with pulse lengths of $t_{\pi/2}$ = 16 ns and t_π = 32 ns and a delay of τ = 300 ns. ENDOR Mims ENDOR was recorded with the pulse sequence $\pi/2 - \tau - \pi/2 - T - \pi/2 - \tau - echo$ with $t_{\pi/2}$ = 16 ns and a 12 µs radiofrequency pulse during the delay T. The power of the radiofrequency pulse was amplified with a Bruker 150 W 10 kHz – 220 MHz RF amplifier and adjusted to obtain a π pulse for a ^1H nucleus. Radio frequencies were varied between 5 MHz below and 5 MHz above the proton Larmor frequency and varied stochastically so as to avoid RF-heating of the sample. The delay τ was varied between 150 and 330 ns in 60 ns increments and spectra were added together to account for blind-spots that arise in Mims ENDOR measurements. ENDOR spectra were recorded at the field position with maximum intensity (corresponding to $g_\perp$). Field-dependent PEANUT experiments were recorded using the pulse sequence $\pi/2 - \tau - p_x - p_{-x} - \tau - echo$ with $t_{\pi/2}$ = 16 ns and τ = 200 ns. The p_x pulse was varied between 2048 ns and 6144 ns in 16 ns increments and the p_{-x} pulse correspondingly was varied between 6144 ns and 2048 ns. The initial length of the p_x pulse was chosen to only record the central region of the PEANUT trace involving the rotary echo and avoid the nutation decay signal at early and late pulse lengths. The field-dependent PEANUT spectrum was recorded with a field resolution of 0.1 mT.

1.3 Deconvolution of Reduction Titrations

Singular value decomposition (SVD) was attempted to estimate the number of components in a reduction titration but due to the similarity of the signals no clear cut-off could be determined. Instead, the number of components was estimated manually based on the expected number of open-shell species and the number of local maxima in the signal intensity profiles of the titrations. The initial guess spectra for the deconvolution algorithm were selected by picking spectra from the titration series which approximately corresponded to a certain oxidation state (e.g. **P*N*$^{3-}$** at 3 equivalents of reducing agent). The EPR spectra of the individual components were obtained by multivariate curve resolution (MCR) using a home-written script, in which the concentration profiles and spectral components were optimized in an alternating manner using a least-square fitting algorithm. The experimental 2D spectrum is attempted to be reconstructed by first optimizing the concentration coefficients while keeping the guess-spectra constant and subsequently optimizing the spectra while keeping the concentration profiles constant. These two steps were repeated 50 times or until the process converged. After each iteration, any negative concentrations were set to 0. Due to the presence of EPR-silent oxidation states, it was not possible to constrain the sum of the relative concentrations to one. After the optimization loop, the concentration profile of each species was corrected by scaling it by the double integral of its deconvoluted spectrum to ensure the correct relative proportions of the concentration profiles and obtain the correct relative concentrations. In addition, all relative concentrations were rescaled by a common factor to ensure that the sum of all relative concentrations was ≤1. The relative concentration of EPR-silent species was estimated by summing the relative concentrations of the EPR-active species and subtracting this sum from 1 for each point in the titration. This relied on the assumption that the relative concentration of the highest reduced oxidation state was 1 at the end of a titration (the final state was a signal intensity maximum in all cases).

1.4 Computational Methods

DFT Geometry Optimizations Structures of the porphyrin oligomers were optimized at the PBE0+D3(BJ)/def2-SVP(H, C, N)/def2- TZVP(Zn) level of theory using Gaussian 16 with a tight convergence threshold. Frequency calculations were attempted to confirm minima but were not successful due to the large system size. All calculations were done with phenyl side chains instead of the bulky 3,5-bis(trihexylsilyl)phenyl side chains to speed up computation times. Calculations of EPR parameters were carried out on the optimized structures in ORCA 5.0. Different functionals based on the PBE exchange and correlation functionals were used. The functionals were modified by manually setting the amount of constant exact exchange (ACM-A parameter) and/or the range-separation parameter ω. If range-separation and constant exact exchange were used in the same calculation, the parameter describing the portion of range-separation was set to 1 – ACM-A. The EPR-II basis set was used for hydrogen atoms, def2-SVP on carbon and nitrogen atoms and def2-TZVP on Zn atoms.

^{1}H ENDOR spectra were simulated using the saffron function in *EasySpin*. ENDOR spectra for different hydrogen nuclei were simulated separately and summed up subsequently with no further modification of their relative intensities. Simulations were done with the values of τ used in the experiments and with *g*-tensors obtained from field-sweep experiments. The magnitudes and orientations of hyperfine coupling tensors were directly obtained from the DFT output files. The ENDOR linewidth was adjusted to optimize the fit between experiment and simulations and ranged between 0.4 and 0.6 in all cases.

The MATLAB code snippet below gives an outline of how the ENDOR spectra were simulated based on the DFT hyperfine coupling tensors and the experimental parameters:

```
%% Experiment setup
Exp.Sequence = 'MimsENDOR';
Exp.nPoints = 401;
Exp.mwFreq = 33.90787;
Exp.ExciteWidth = 1/64 * 1e3;
Exp.Field = 1210.9;
Exp.Range = [larmorfrq('1H',Exp.Field)-5.5
larmorfrq('1H',Exp.Field)+5.5];

%% System setup
Sys.g = [...]; % g-tensor was obtained from fitting to experimental
echo-detected field-sweep
% note: the g-tensor is well-aligned with the molecular frame and
was set accordingly
Sys.n = 1;
Sys.Nucs = '1H';
A_tensors= [...] % Nx3x3 array containing all hyperfine coupling
tensors included in the simulation
nb_HF = size(A_tensors,1);
Sys.AFrame = [0 0 0]*pi/180; % frame of each hyperfine coupling is
set implicitely as A tensors contain orientaion information
Sys.lwEndor = ...; % this was optimised (constrained between 0.4 and
0.8)

%% Simulation loop
spectra_individual = zeros(nb_HF,Exp.nPoints);
for ii = 1:nb_HF
    Sys.A = reshape(A_tensors(ii,:,:), 3, 3);
    %Sys.lwEndor = ENDOR_lw(ii);
    spc_sim_individual = endor_simulation(Sys, Exp);
    spectra_individual(ii,:) = spc_sim_individual;
end
spectra_all = sum(spectra_individual, 1);


%% Functions
function sim_spectrum = endor_simulation(Sys,Exp)
    spectra=zeros(4,Exp.nPoints);
    tau = [0.150 0.210 0.270 0.330];
    for j = 1:4
        Exp.tau = tau(j);
        spectra(j,:) = saffron(Sys,Exp);
    end
    sim_spectrum = sum(spectra,1);
end
```

2. Electrochemistry

Square-wave voltammograms of **P*N*** (*N* = 1–6) and $CoCp^*_2$ relative to Fc/Fc^+, measured under an inert atmosphere, are shown in **Figure S*1*** (reproduced with data published by *Hergenhahn et al.* [2]).

The successive reduction peaks are only resolved up to **P3**, after which the peaks begin to be too closely spaced. While the nth reduction peak shifts to more negative potentials, the one-electron reducing agent $CoCp^*_2$ (E = –1.86 Vvs. Fc/Fc^+) is sufficiently strong to reduce any of the measured porphyrin oligomers **P*N*** to their $\mathbf{P}\boldsymbol{N}^{N-}$ polyanion.

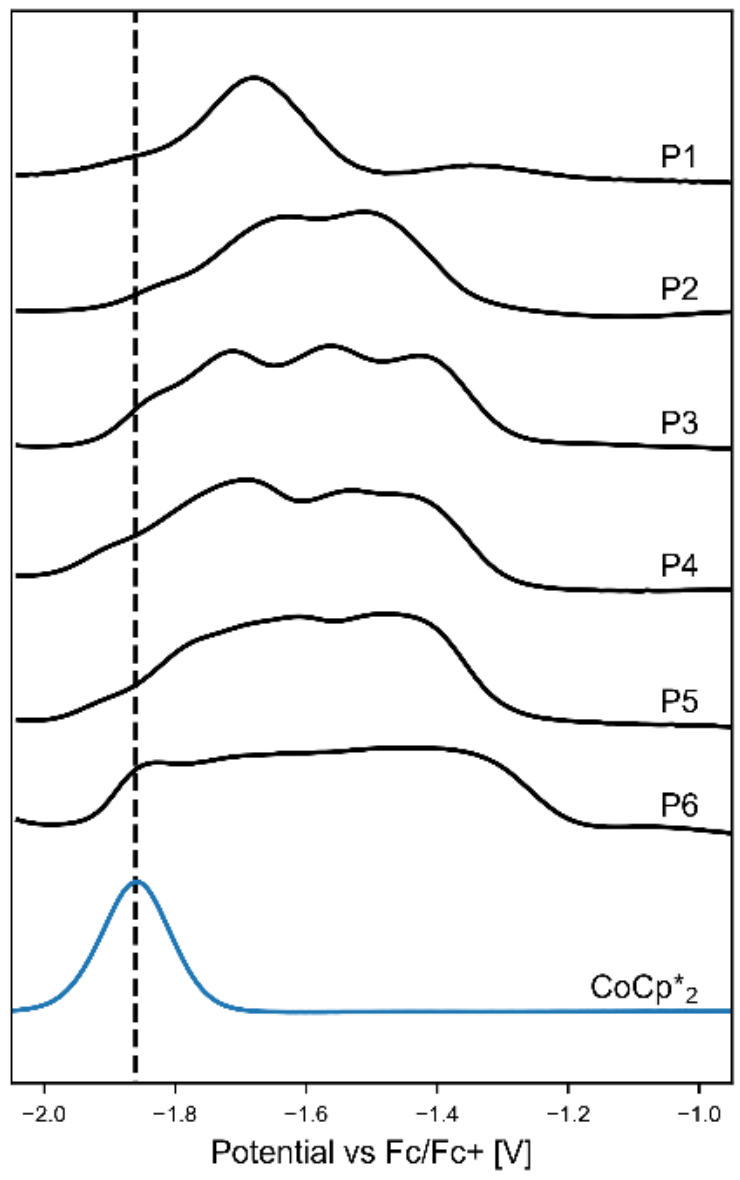

Figure S1 Square wave voltammetry of PN (N=1-6) and $CoCp^*_2$ recorded in THF with 0.1 M [Bu4N][PF6].

3. CW-EPR Signal Stability

The stability of the room temperature EPR signal was tested for the porphyrin monomer monoanion $\mathbf{P1}^{1-}$ by measuring the CW-EPR as shown in Figure S2. The signal intensity (relative to t=0) was determined from the double integral of the EPR signal. The half live was determined by fitting an exponential decay function giving a $t_{1/2}$ of about 5 hours, longer than the time scale of the EPR titration (>1h). Also, the EPR signals of the anions of longer oligomers were generally found to be more stable than that of the monomer anion.

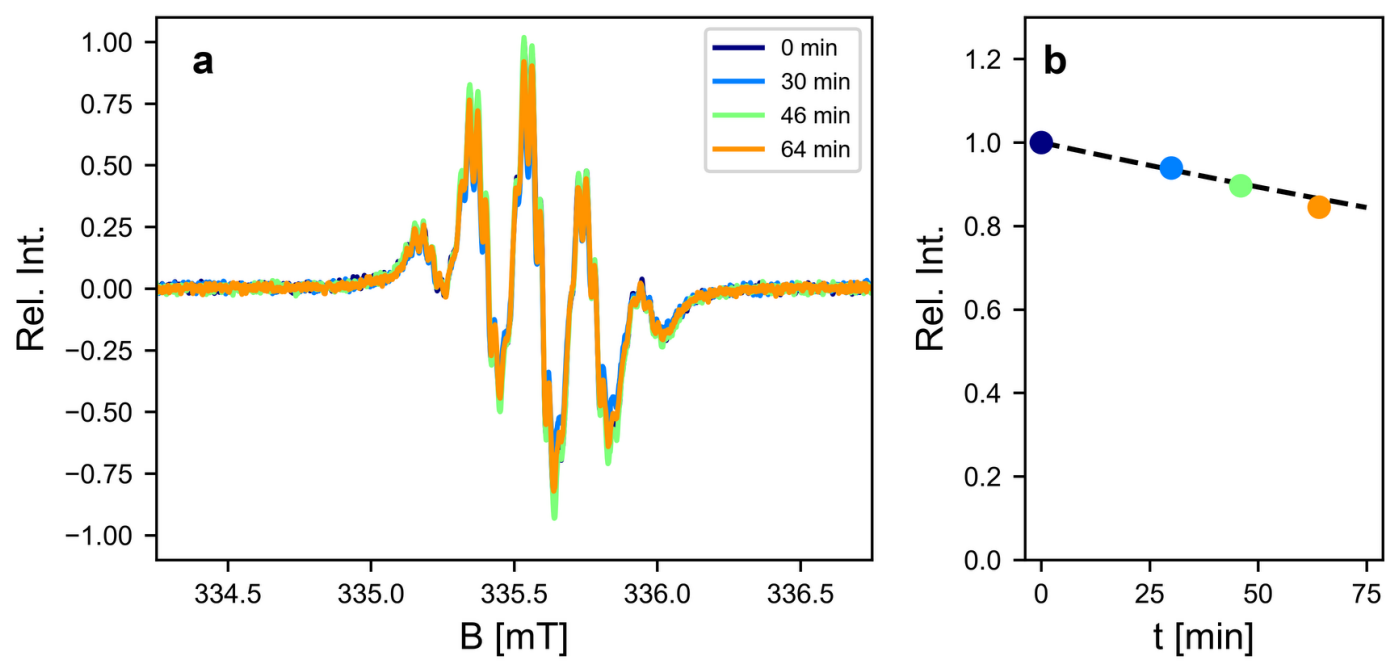

Figure S2 a) Spectra recorded at different time points. b) Relative intensity over time with fitted exponential decay ($t_{1/2}$ = 306 min).

4. Optimal number of components for P7 reduction titration

Unlike for the reduction titrations of **P3** and **P5**, the number of maxima in the signal intensity in **P7** did not correspond to the number of expected open-shell species. Therefore, it was not clear what number of components should be used in the deconvolution of the EPR data. **Figure S3** shows the deconvoluted spectra for different numbers of components as well as the corresponding relative concentration profiles and the associated RMSDs. If only the odd-numbered oxidation states were EPR-active, there should be four species in the titration of **P7** (1-, 3-, 5- and 7- states). However, this results in unreasonable concentration profiles that require some of the species to be formed, depleted and formed again, which is unlikely in a reduction titration. Including an additional component (i.e. 5 EPR-active components) mostly resolves this issue with all species having a single distinct maximum in their concentration profile. The points, at which these species appear in the titration, suggests the most likely assignment as **P7**$^{1-}$, **P7**$^{2-}$, **P7**$^{3-}$, **P7**$^{5-}$, **P7**$^{7-}$; in addition, the resulting concentration of EPR-silent species has 3 maxima which would naturally correspond to **P7** (neutral), **P7**$^{4-}$ and **P7**$^{6-}$. The RMSD is not significantly reduced by including further components. Also, since any additional components do not clearly account for specific oxidation states, selecting five components were chosen as the best compromise between quality of fit and chemical meaningfulness of the components.

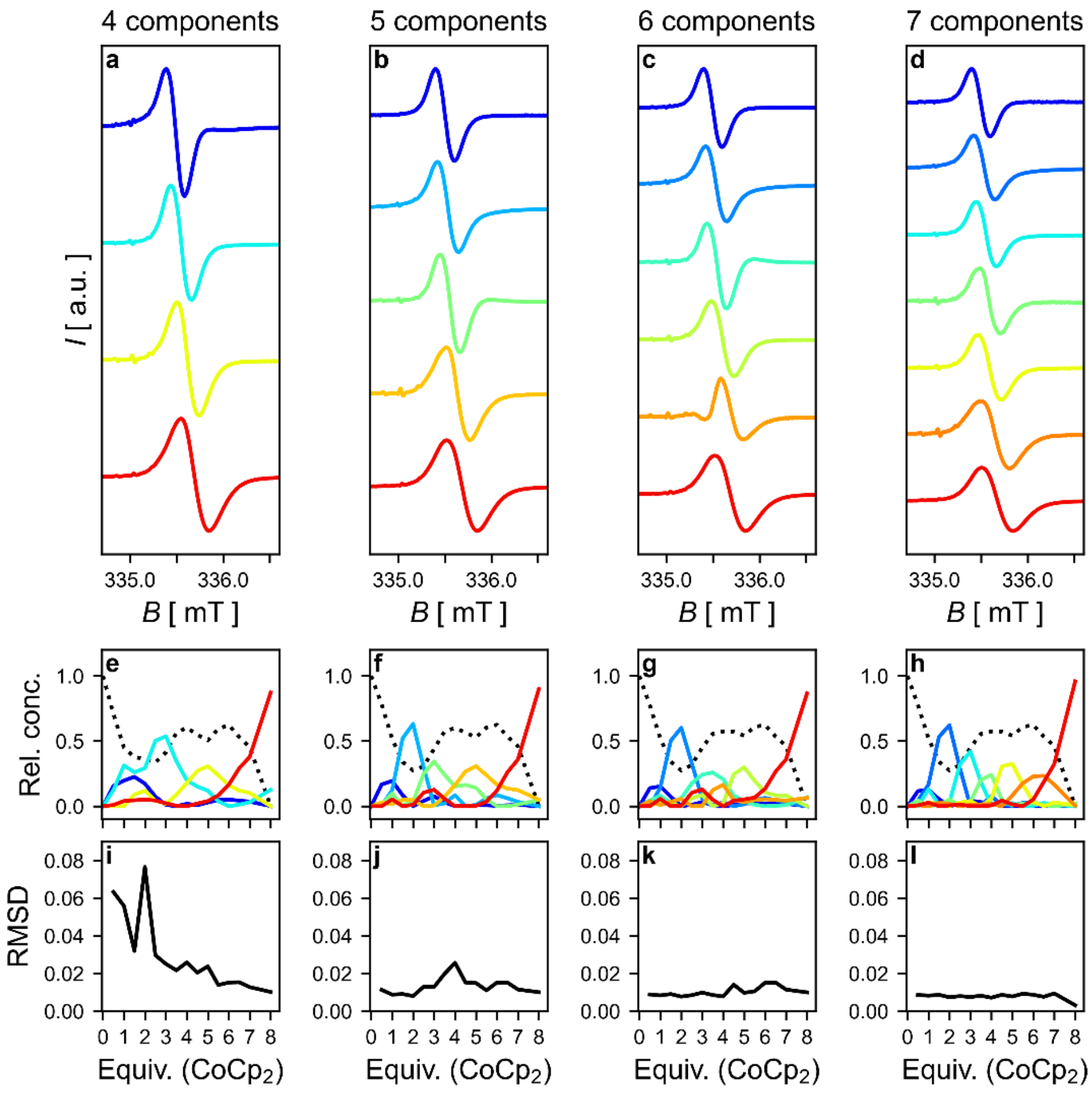

Figure S3 a) to d) Deconvoluted components of the CW-EPR titration of **P7** for different number of components. e) to h) Relative concentrations of the components during the EPR titration. The black, dotted line gives difference between 1.0 and the sum of relative concentrations and indicates the relative concentration of EPR-silent species. i) to l) RMSD between the experimental spectrum and the recreated spectrum from the deconvoluted spectra.

5. CW-EPR spectral simulations

Least-square fitting of CW-EPR simulations was done using *EasySpin* to determine the *g*-factors, line shapes and hyperfine coupling constants of the components found in the reduction titrations. No constraints were applied to the numerical simulations. The resulting simulations are shown in **Figure S4** and the corresponding simulation parameters in **Table S1**.

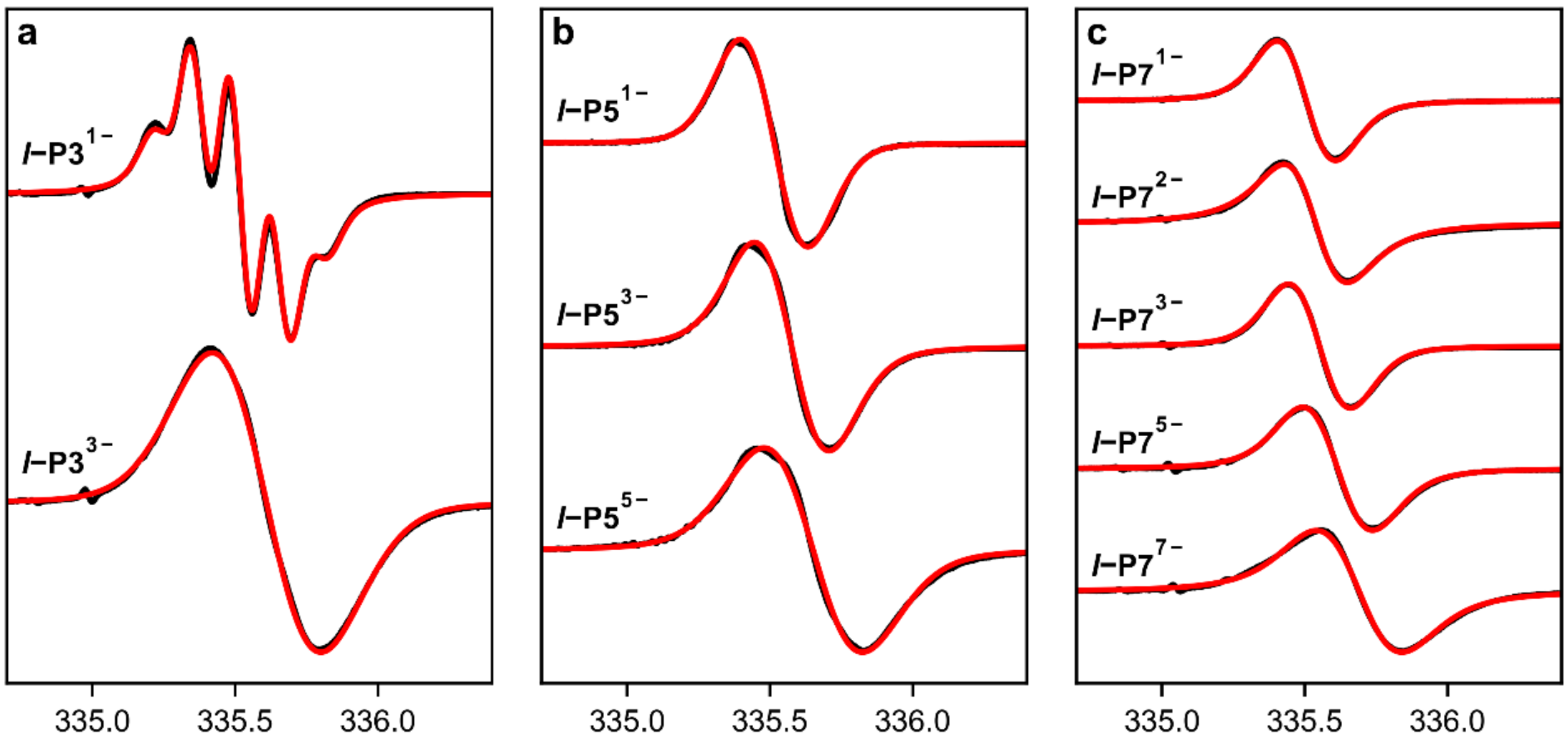

Figure S4 Experimental CW-EPR spectra obtained from deconvolution of reduction titration experiments at room temperature (black) and numerical simulations (red).

Table S1 CW-EPR simulation parameters.

Structure	g	Gaussian Linewidth Component [mT]	Lorentzian Linewidth Component [mT]	Nuclei	*N*	A_{iso}
**P3^{1-}**	2.0017	0.075	0.058	H	4	3.71
**P3^{3-}**	2.0011	0.354	0.051			
**P5^{1-}**	2.0017	0.22	0.032			
**P5^{3-}**	2.0013	0.221	0.072			
**P5^{5-}**	2.0009	0.303	0.077			
**P7^{1-}**	2.0017	0.163	0.074			
**P7^{2-}**	2.0016	0.106	0.17			
**P7^{3-}**	2.0015	0.178	0.069			
**P7^{5-}**	2.0011	0.186	0.1			
**P7^{7-}**	2.0007	0.203	0.16			

6. Additional Pulsed EPR Data

The echo-detected field-sweep spectra of the polyanions **P*N***$^{N-}$ (*N* = 3,5,7) and their monoanion counterparts **P*N***$^{\bullet-}$ are shown in **Figure S5**. The polyanions have similar EPR-spectra to the monoanions consisting of an axial *g*-tensor and no resolved hyperfine pattern. The *g*-anisotropies in **P*N***$^{N-}$ are larger than in **P*N***$^{\bullet-}$. The larger *g*-anisotropy is consistent with the population of higher lying orbitals as more electrons are added. The energies of these orbitals are more closely spaced, which leads to a smaller energy gap to excited electronic states that contribute towards the *g*-anisotropy ($\propto 1/\Delta E$).

Given the presence of multiple additional electrons in the π-system of the conjugated porphyrin oligomers **P*N***, it is interesting to consider the potential presence of higher spin multiplicities. The spin state of the polyanions states was investigated using field dependent PEANUT (phase-inverted echo-amplitude detected nutation) experiments, which have previously been used to study anionic porphyrin systems with several spin multiplicities. The Fourier transforms of the PEANUT field sweeps for **P*N***$^{N-}$ and **P*N***$^{\bullet-}$ (*N* = 3, 5, 7) are shown in **Figure S6**. The spectra of the radical monoanions show a single signal at a nutation frequency at 5.0 MHz and serve as a reference for a spin-1/2 system. The spectra of the polyanionic systems also show a single dominant nutation frequency at the same frequency as the monoanions indicating that they are also present in doublet states.

The large number of hydrogen nuclei that contribute to the ^{1}H ENDOR spectra of **P*N***$^{N-}$ and **P*N***$^{\bullet-}$ result in an irregular shape which makes a quantitative determination of the width of the spectrum difficult (as the full width at half maximum employed in previous studies is not a good metric). Therefore, the width of an ENDOR spectrum was defined by its turning points as defined by the maxima and minima of its derivative. The spectral widths were determined by the difference between the first maximum and the first minimum of the derivative as shown in **Figure S8**.

Reduction of the porphyrin oligomers to their highly charged states leads to a change in the spin memory time T_m as shown in **Figure S9** for the monoanions and polyanions of **P*N*** (*N* = 3,5,7). The T_m times were determined by fitting stretched exponential decay functions to experimental echo decay spectra. The decrease in spin memory time is more pronounced for the larger, more negatively charges systems as can be seen from the ratios of T_m determined for **P*N***$^{N-}$ and **P*N***$^{\bullet-}$ as shown in **Table S2**.

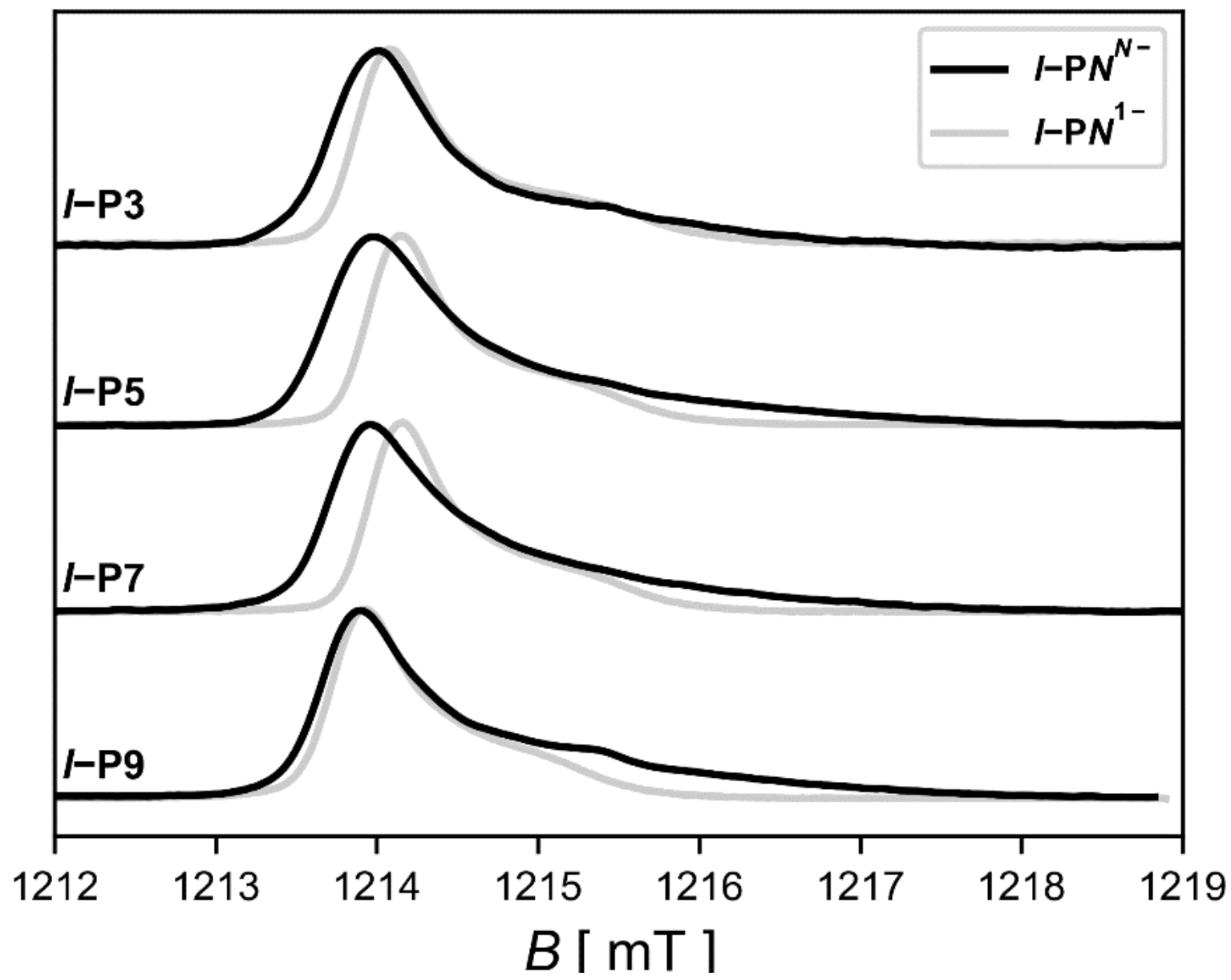

Figure S5 Echo-detected field-sweep spectra of **P*N***$^{N-}$ (*N* = 3, 5, 7, 9) recorded at 80 K at Q-band frequencies. Spectra of the monoanions **P*N***$^{\bullet-}$ (*N* = 3, 5, 7) are shown in grey for comparison.

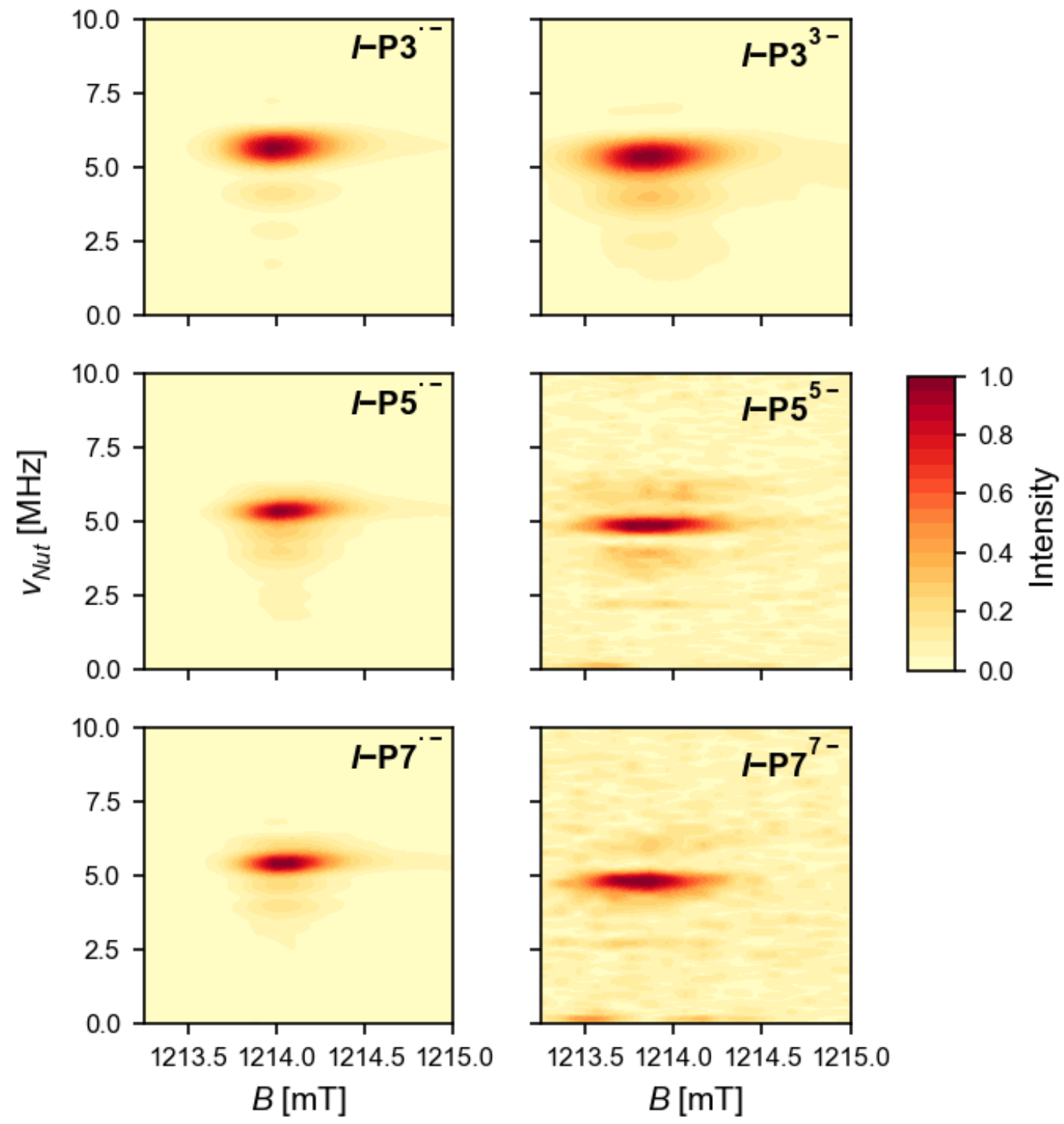

Figure S6 Fourier transforms of PEANUT nutation field sweep experiments of **P*N***$^{\bullet-}$ and **P*N***$^{N-}$ (*N*=3, 5, 7) recorded at Q-band frequencies at 80 K.

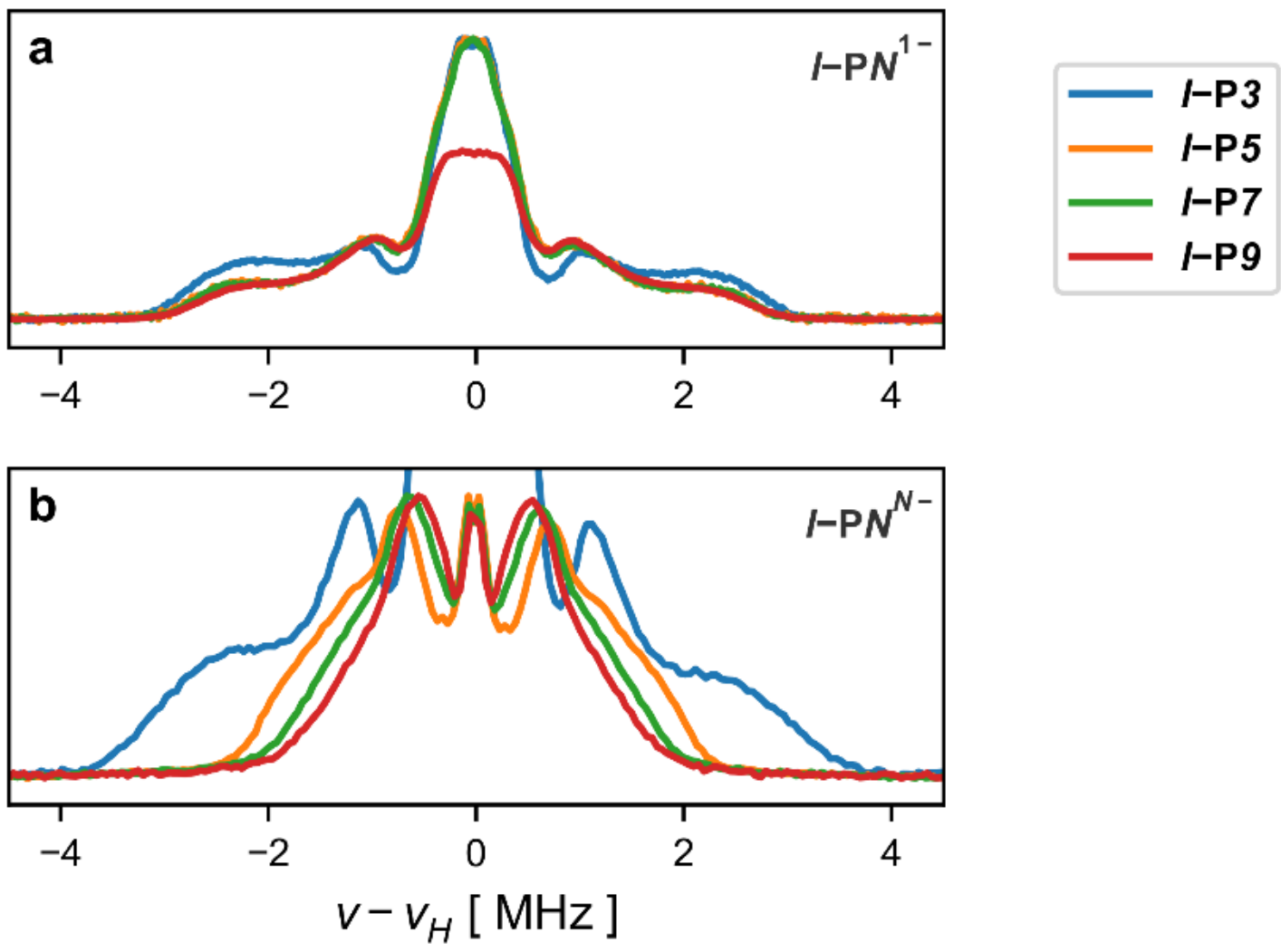

Figure S7 ^{1}H ENDOR spectra of a) ***PN***$^{1-}$ and b) ***PN***$^{N-}$ (*N* = 3, 5, 7) recorded at field positions corresponding to $g_{\perp}$ at 80 K at Q-band frequencies. Spectra were overlayed for easier comparison.

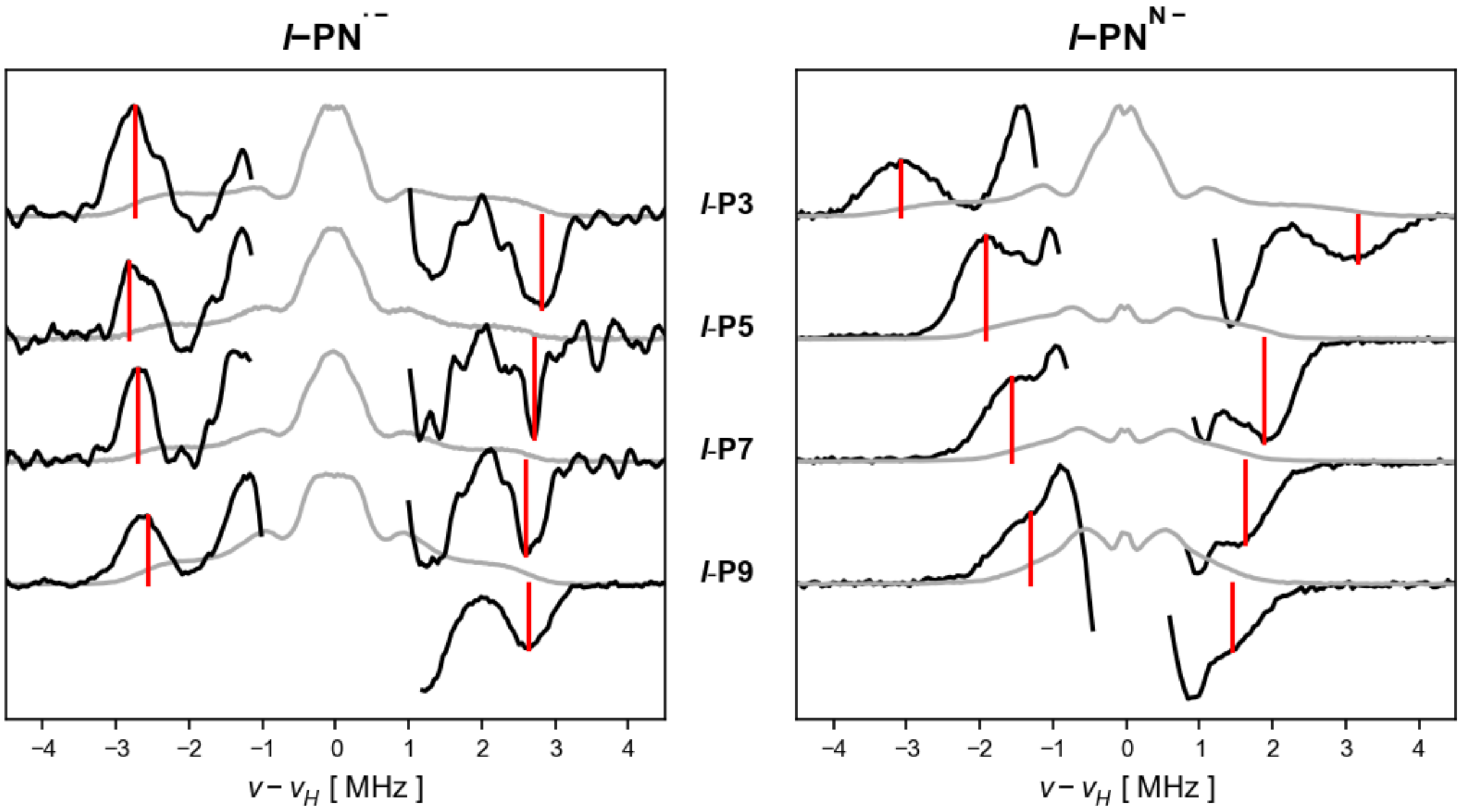

Figure S8 Derivative of ENDOR spectra of ***PN***$^{\bullet-}$ and ***PN***$^{N-}$ (*N*=3, 5, 7, 9) recorded at Q-band frequencies at 80 K. The spectral width was determined from the first maximum and last minimum of the derivative, corresponding to the turning points of the ENDOR spectrum.

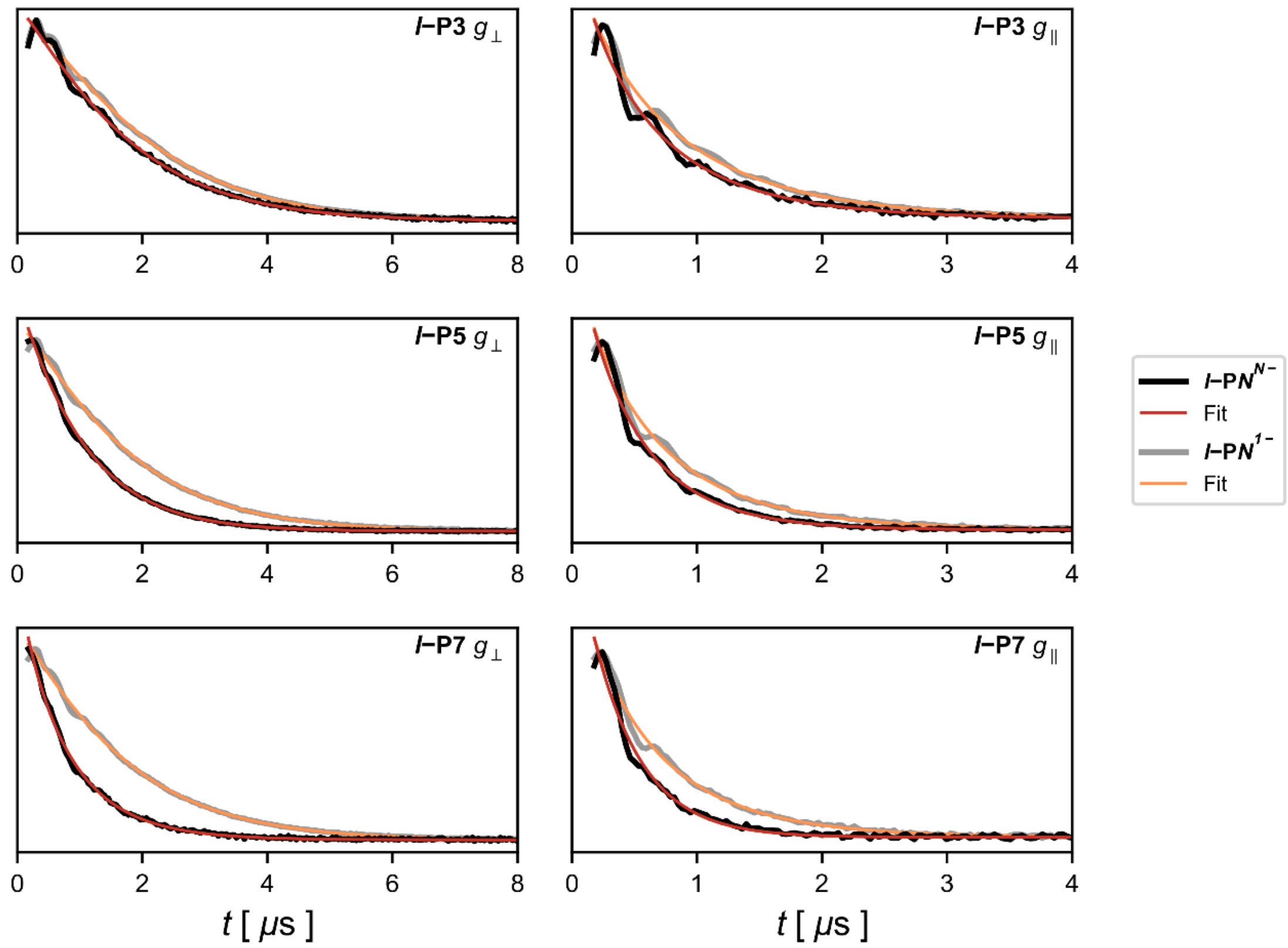

Figure S9 Echo decay experiments of **P***N*$^{N–}$ (N = 3, 5, 7) recorded at 80 K at Q-band frequencies at field positions corresponding to $g_\perp$ (left) and $g_\parallel$ (right). Spin memory times T_m were determined by fitting stretched exponential decays (red). Echo decay experiments for the monoanions **P***N*$^{•–}$ (N = 3, 5, 7) are shown for comparison (experiment in grey and fit in orange)

Table S2 Spin memory times T_m of **P***N*$^{•–}$ and **P***N*$^{N–}$ (N = 3,5,7) in µs.

Structure	Orientation	P*N*$^{•–}$	P*N*$^{N–}$	Ratio
**P3**	$g_\perp$	2.16	1.81	0.84
	$g_\parallel$	0.713	0.54	0.76
**P5**	$g_\perp$	1.81	1.07	0.59
	$g_\parallel$	0.56	0.45	0.80
**P7**	$g_\perp$	1.77	0.73	0.41
	$g_\parallel$	0.549	0.40	0.72

7. Additional Data for Density Functional Theory Calculations

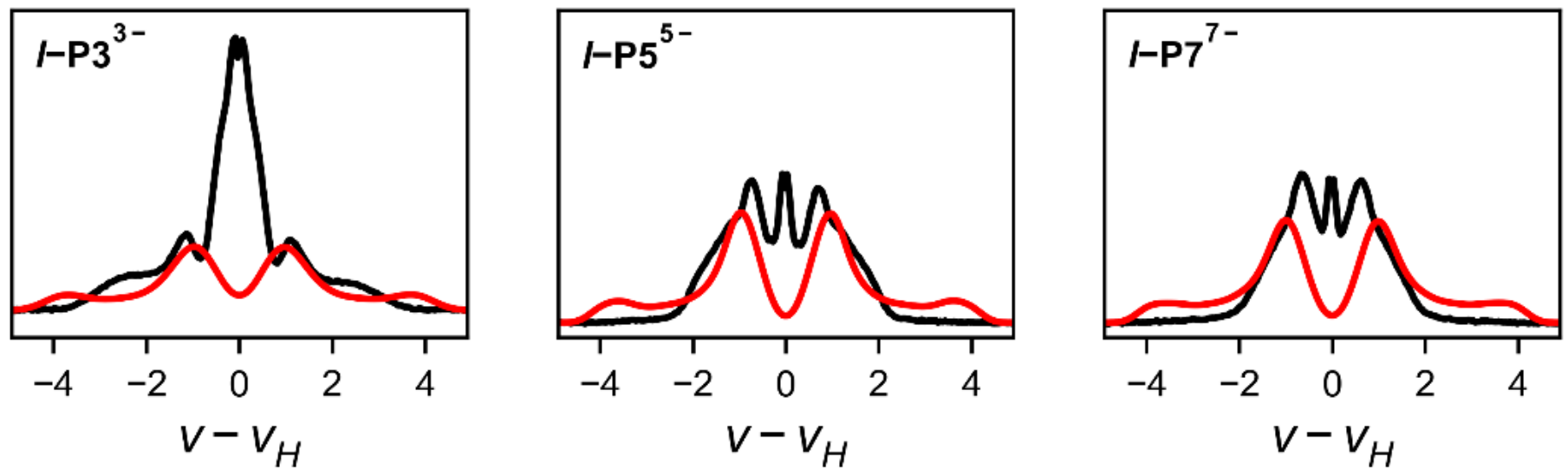

Figure S10 Experimental ^{1}H ENDOR spectra of **P*N*$^{N-}$** (black) and initial unsuccessful simulations (red). The simulations were based on hyperfine tensors from DFT calculations using the lc-ωPBE functional and the range-separation parameter ω that gave the best fit for the monoanion systems (ω = 0.175) but overestimate the hyperfine coupling interactions in **P*N*$^{N-}$**.

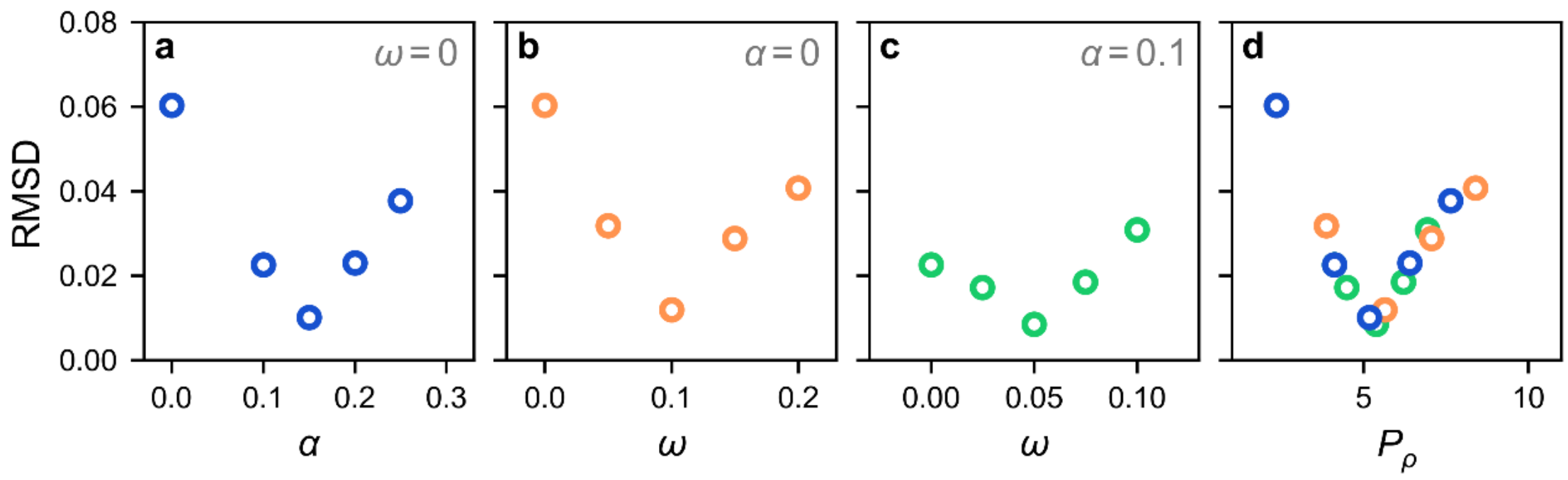

Figure S11 Root mean square deviation (RMSD) between experimental ^{1}H ENDOR spectrum of **P3^{3-}** and simulations based on hyperfine coupling constants derived from DFT calculations. a) RMSD for PBE based hybrid functional with different amounts of exact exchange α. b) RMSD for range-separated functional lc-ωPBE with different range separation parameters ω. c) RMSD for adjusted range-separation functional based on lc-ωPBE but with a constant amount of exact exchange of α = 0.1. d) RMSD as a function of spin polarisation.

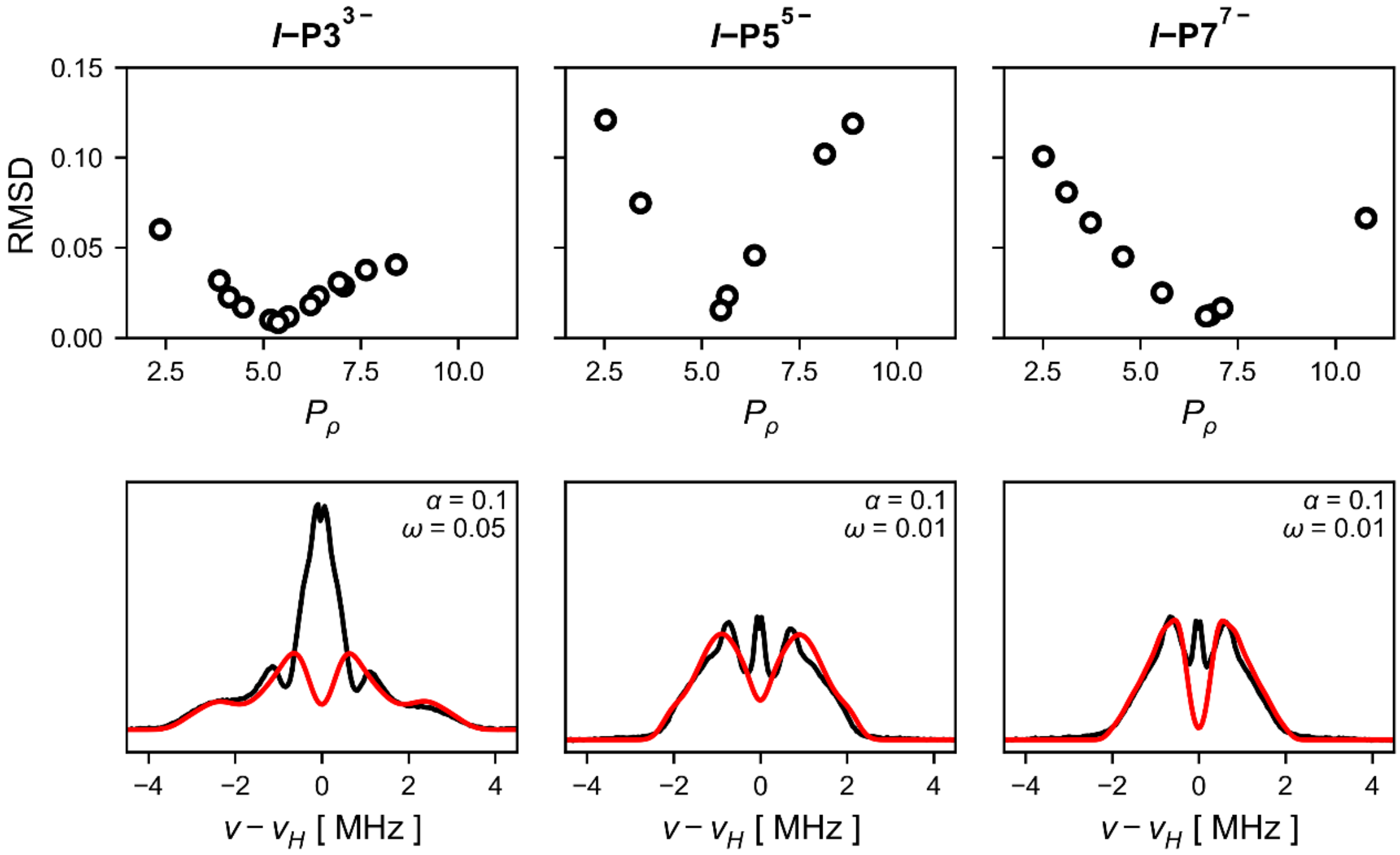

Figure S12 (Top) Root mean square deviation (RMSD) between experimental ^{1}H ENDOR spectra of **P*N*$^{N-}$** and simulations based on DFT calculated hyperfine coupling constants for different amounts of spin polarisation. (Bottom) Experimental ^{1}H ENDOR spectra of **P*N*$^{N-}$** (black) and best fit simulation (red, DFT functional parameters are given in figure). The central peak was excluded from the RMSD calculation in all systems.

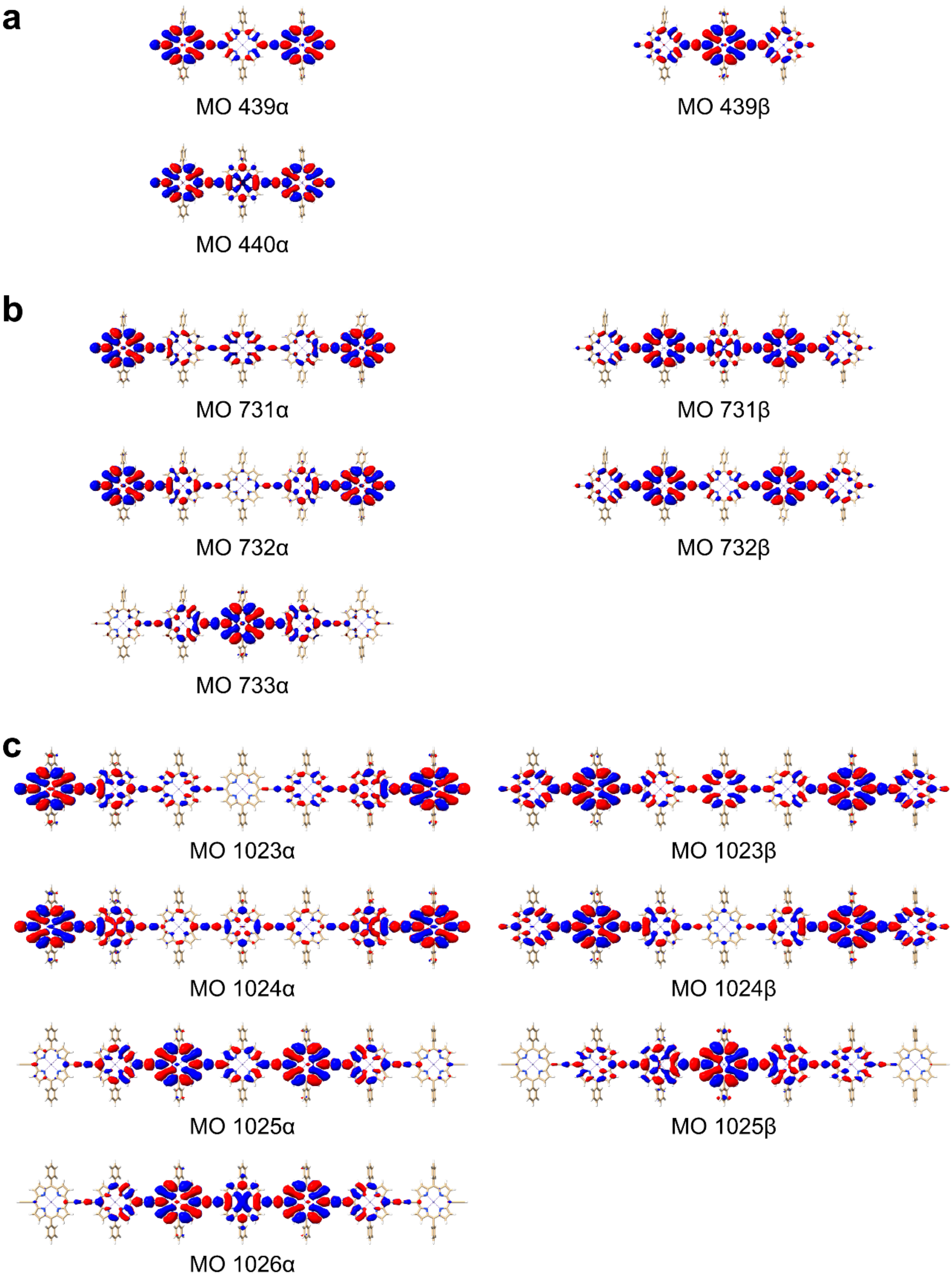

Figure S13 Highest occupied molecular Kohn-Sham orbitals obtained from DFT calculations for the wavefunction that resulted in the spin density and hyperfine coupling tensors in best agreement with experimental ^{1}H ENDOR spectra for a) **P3**$^{3-}$, b) **P5**$^{5-}$ and c) **P7**$^{7-}$.

8. References

[1] Tait, C. E.; Neuhaus, P.; Peeks, M. D.; Anderson, H. L.; Timmel, C. R. *J. Am. Chem. Soc.* **2015**, *137*, 8284

[2] Hergenhahn, J.; Holmes, J. M. ; Deng, J.-R.; Gotfredsen, H.; Jacobs, R. M. J.; Kopp, S. M.; Timmel, C. R.; Anderson, H. L. *J. Am. Chem. Soc.* **2025**, *147*, 978
